# Effect of Dietary Fiber Intake on Chronic Low-Grade Inflammation in Children and Adolescents: A Systematic Review and Meta-analysis of Randomized Controlled Trials

**DOI:** 10.1016/j.cdnut.2025.107511

**Published:** 2025-07-24

**Authors:** Mª Isabel Benedicto-Toboso, Andressa Freire Salviano, María L Miguel-Berges, Isabel Rueda-De Torre, Luis A Moreno, Alba M Santaliestra-Pasías

**Affiliations:** 1 Growth, Exercise, Nutrition and Development (GENUD) Research Group, Departamento de Fisiatría y Enfermería, Universidad de Zaragoza, Zaragoza, Spain; 2 Instituto Agroalimentario de Aragón (IA2), Universidad de Zaragoza-CITA, Zaragoza, Spain; 3 Instituto de Investigación Sanitaria de Aragón (IIS Aragón), Zaragoza, Spain; 4 Centro de Investigación Biomédica en Red de Fisiopatología de la Obesidad y Nutrición (CIBERObn), Instituto de Salud Carlos III, Madrid, Spain; 5 Faculdade de Saúde Pública, Universidad de São Paulo (FSP/USP), Brazil

**Keywords:** chronic low-grade inflammation, dietary fiber, inflammatory markers, C-reactive protein, IL-6, TNF-α, children, adolescents, systematic review, RCTs

## Abstract

Previous research suggests that dietary fiber (DF) intake may help reduce chronic low-grade inflammation (CLGI), a condition linked to the early development of cardiometabolic risk factors. Childhood and adolescence represent critical periods for preventing noncommunicable diseases, when adopting healthy eating habits, including adequate fiber consumption, could effectively control CLGI. However, the evidence on DF’s impact on CLGI in the pediatric population remains inconsistent and has not been comprehensively reviewed in a single article. Therefore, we aimed to conduct a systematic review and meta-analysis to assess the effect of DF intake on CLGI in children and adolescents. A systematic search was performed in 4 databases up to January 2025. Two reviewers screened 2030 studies based on inclusion criteria: randomized controlled trials involving participants ≤18 y, interventions (Is) with any type of DF (supplementation, fiber-rich foods, or fiber intake advice) and reporting serum CLGI markers, including C-reactive protein (CRP), interleukin (IL)-6 and TNF-α, among others. Twenty-five randomized controlled trials were included in the systematic review, which showed that DF may have beneficial effects on CRP, IL-10, adiponectin, IL-1β, and IL-6 concentrations; though findings were inconsistent, with some studies reporting no significant changes. Meta-analysis was conducted for CRP, IL-6, and TNF-α. Meta-analysis for CRP concentrations included 10 studies and revealed a significant decrease following DF Is compared to controls (mean difference: –0.640; 95% CI: –1.075, –0.204). Meta-regression revealed that Is based on fiber supplementation resulted in significantly greater CRP reductions compared to those involving fiber-rich foods. Meta-analysis for IL-6 and TNF-α concentrations showed no significant effect after DF I. In conclusion, this review provides evidence that fiber Is may have a beneficial impact on certain markers of CLGI in children and adolescents, particularly by reducing serum CRP concentrations. However, the findings also reveal inconsistencies in the effects of fiber intake on other inflammatory markers.

This trial was registered at PROSPERO as CRD42024516794.

## Introduction

Chronic low-grade inflammation (CLGI) is a persistent, moderate inflammatory process characterized by the overproduction of acute-phase proteins, outstanding C-reactive protein (CRP), and pro-inflammatory cytokines such as IL-6 and TNF-α [1]. CLGI has been linked to the early development of cardiometabolic risk factors, including obesity [2], insulin resistance, and metabolic syndrome [3,4] even in childhood and adolescence. Excess adiposity, a growing concern in the pediatric population, is also associated with increased concentrations of inflammatory markers [5]. Global estimates indicate that 37 million children under 5 are overweight/obese, conditions strongly linked to CLGI [6]. Given the importance of childhood as a critical developmental period for growth and health trajectory, controlling noncommunicable diseases’ risk factors, such as CLGI, during this phase is crucial. Prevention through promotion of healthy habits, including a healthy diet and physical activity, is emerging as an effective strategy for controlling CLGI [[7], [8], [9]].

Dietary fiber (DF) is a fundamental component of a healthy diet. A high-fiber intake has many health benefits, beyond its local effects in the gut to systemic impact, including the reduction of chronic inflammation [10]. In the gastrointestinal tract, it has functional effects such as promoting satiety, stool bulking, and improving motility [11,12]. Certain types of fiber, such as psyllium, glucomannan, guar gum, and lignin, are particularly renowned for having these properties [13]. Nonfermentable or poorly fermentable fiber, predominantly insoluble, is the 1 that primarily confers the previously mentioned benefits. Additionally, at the intestinal level, soluble, viscous fibers like β-glucans reduce dietary cholesterol absorption and bile acids reabsorption [14]. DF also has a physiological systemic impact through its fermentation by the gut microbiome. The resulting short-chain fatty acids, such as acetate, propionate, and butyrate, promote the growth of beneficial bacteria [15,16]. These prebiotic effects are well established for fermentable fibers such as fructooligosaccharides (FOS) [17], inulin [18], and acacia gum [19]. Adequate fiber intake also decreases gut-derived uremic toxins and inflammation [20]. Recent research shows that fiber consumption has a beneficial effect on the immune system [16]. In adults with obesity, inulin-type fructans have shown positive effects on low-grade inflammation [21]. Additionally, a fiber-rich diet may also modify the production of inflammatory cytokines, particularly TNF-α and IL-6, through changes in the gut microbiota [22]. Insufficient fiber intake has been associated with immune-related disorders in children [10,23].

Nutritional guidelines recommend an intake of 14 g of fiber per 1000 kcal from early childhood [[24], [25], [26], [27]]. However, fewer than 3% of children meet these recommendations [[28], [29], [30], [31]]. An adequate fiber intake appears to be essential for controlling CLGI, as previous studies suggested that DF consumption lowers inflammatory markers [[32], [33], [34]]. A systematic review showed that most observational studies in adults reported an inverse association between DF intake and IL-6 and high-sensitivity CRP (hs-CRP) concentrations [33]. Another systematic review of randomized controlled trials (RCTs) in adults with obesity has also shown that DF intake is related to a reduction in CRP concentrations [32].

In children and adolescents, observational studies suggested an inverse association between DF and CLGI [[35], [36], [37], [38]]. However, the evidence from observational studies is not as strong as that from RCTs. Recently, several trials have assessed the effect of DF intake on CRP concentrations and other CLGI markers. Therefore, we aimed to conduct a systematic review, bringing together the current evidence from interventional (I) studies investigating associations between DF intake and markers of low-grade inflammation, including CRP, IL-6, and TNF-α, among others, in both children and adolescents. Our study hypothesizes that DF intake has a beneficial effect on CLGI markers in children and adolescents.

## Methods

This systematic review was conducted based on the Cochrane Handbook for Systematic Reviews and Is methodology [39] and following the PRISMA to ensure the comprehensive capture of all recommended information within the article [40]. A detailed protocol was developed a priori and registered in PROSPERO (CRD42024516794) to enhance transparency and reduce risk of bias. All systematic review steps were performed in duplicate by 2 independent researchers (MIB-T and AFS).

### Search strategy

A systematic search was performed in 4 databases: PubMed, Web of Science, Scopus, and the Cochrane Central Register of Controlled Trials. There was no restriction on publication language and date up to 21 March, 2024. The databases’ email alert service was used to update our search up to 30 January, 2025. The specific search strategies for each database are attached in the supplementary material (Supplemental Tables 1 and 2). In brief, the following search terms combination was used: [DF terms OR (DF type terms AND consumption/supplementation terms) – as I terms] AND (CLGI/ inflammation markers synonyms – as outcome terms) AND (child and adolescent synonyms – as population terms) AND (RCT – as study type terms).

Additionally, a hand-search was undertaken to retrieve further relevant studies by reviewing the references of the selected articles either manually or with the assistance of the tool Research Rabbit and by consulting clinicaltrials.gov and the WHO International Clinical Trials Registry Platform (ICTRP) to identify unpublished and ongoing trials. These trial registers are a valuable source of information about unpublished completed and ongoing trials. Information about protocols of potentially relevant ongoing trials was compiled in Supplemental Table 3.

### Peer review

Search, title, abstract, and full-text screening, data extraction, and quality assessment were conducted in duplicate by 2 review authors (MIB-T and AFS) separately, independently, and in a standardized way. Any disagreement was discussed until consensus was reached, or by consulting a third author (LAM), or by group discussion.

### Eligibility criteria and study selection

Studies were included if they met all the following criteria: *1*) RCT, *2*) participants were children or adolescents (≤18 y old), *3*) that consumed or were supplemented with DF (any type of fermentable and nonfermentable fiber) or any fiber as a prebiotic or fiber-rich food or product or received food advice focused on increasing fiber consumption. *4*) Studies that provided information about serum markers of CLGI by I group (any information was retrieved: mean differences (MDs), initial and final values, markers only after I, correlations, text and charts only).

When >1 time point during the I was reported, the data from the longest period were used, not considering data from long follow-up periods after the I finished. Data from the highest dose were used when >1 dose was administered for supplementation. In case of multiple publications with duplicated or overlapped data for the same trial, the article with more detailed information was selected. Multiple reports of the same study that reported different inflammatory markers were identified and linked together.

Exclusion criteria were as follows: *1*) Is based on dietary patterns, in which fiber intake is increased as a result of the general I, and were not included due to the difficulty in isolating the specific effect of fiber from that of other dietary components; *2*) Is with fiber combined with probiotics. These studies were excluded except when the control (C) group consumed the same type and dose of probiotic. The same criterion was applied to supplementation with fiber plus iron. Among the excluded studies, there were also those with participants who were *3*) pregnant or had acute *4*) infectious, *5*) inflammatory, or *6*) allergic processes.

For the inclusion in the meta-analysis, the criteria were stricter: the RCTs had to report the net mean changes of blood markers of CLGI and their corresponding SD or provide data to calculate these values. Meta-analysis was only conducted when there were ≥2 studies implementing the same kind of I.

### Data extraction

Search results were imported by Comma-Separated Values (CSV), merged into an Excel file, and deduplicated before screening. From selected studies, the following data was extracted: authors, year of publication, country where the study was conducted, study design and duration, size of the analyzed sample, participant characteristics including age, sex, health condition, anthropometric features such as BMI (in kg/m^2^); I: type of fiber consumed/supplemented, quality (source) and quantity (dose), outcomes presented as differences in inflammatory marker concentrations after the I between groups, study quality.

For all prespecified outcome data, the mean, median, SD, SE, IQR, or 95% confidence intervals (CIs) that were reported at the beginning and the end of I were extracted for analysis.

To assess the potential confounding effect of energy intake on the association between fiber consumption and clinical outcomes, we extracted data on total (T) energy intake when reported in the included studies. We specifically noted whether the I protocols explicitly targeted energy restriction or whether any changes in caloric intake were observed or reported during the study. This information was used to contextualize the findings and assess the independence of fiber’s effects from changes in energy intake.

### Quality and risk of bias assessment

The Physiotherapy Evidence Database (PEDro) scale was used to measure the quality of each included study. The tool enables the assessment of internal (criteria 2–9) and external validity (criterion 1) and the sufficiency of the statistical information provided (criteria 10–11). Articles with a score between 6 and 10 are considered to have a high methodological quality; those with 4–5 are considered to have a moderate methodological quality; and articles with 0–3 points are considered to have low methodological quality [41]. Risk of bias was independently assessed by 2 reviewers (MIB-T and AFS) using the Cochrane tool Rob-2 [42]. The Rob-2 tool comprises 5 domains: *1*) bias arising from the randomization process, *2*) deviations from intended Is, *3*) missing outcome data, *4*) measurement of the outcome, and *5*) selection of the reported result. Differences in the overall judgment between authors were resolved by discussion to arrive at a consensus.

### Data synthesis and statistical analysis

All analyses were performed using OpenMeta [Analyst] software (version v0.24.1). The overall effect of fiber on CLGI was calculated as the MD between baseline and follow-up, comparing I with the C group. Data reported as median and IQR, SE, or CI were transformed to mean and SD using a standard formula previously described [39,43]. When necessary, outcome units were converted to international units.

Between-study heterogeneity was quantified by the I^2^ statistic [39]. A random-effects model was used to calculate the overall effect size. A prespecified subgroup analysis was conducted to identify potential sources of heterogeneity in net marker changes. Subgroups were defined according to the characteristics of the participants (age, health condition, and baseline inflammatory status) and the I design (duration, quantity, and quality of the fiber consumed). Predefined subgroup analyses were undertaken for categories reported in ≥2 studies per subgroup or variable. Subgroup analyses compared results by age (infants: <2 y of age; children, including preschool child and child: 2–12 y; adolescents: 13–18 y); health condition [healthy; subjects with overweight or obesity; participants with chronic diseases with an inflammatory component (asthma, cancer, celiac disease and type 1 diabetes); baseline CRP (≤3 mg/L; >3mg/L), I duration (short-term: ≤8 wk; medium-term: >8 wk, with no long-term, as the maximum duration was 18 wk]; type of DF [fiber supplementation, e.g.,: galactooligosaccharides (GOS), oligofructose-enriched inulin, FOS; fiber-rich foods or products, e.g.,: chia seeds, whole grains (WGs), etc.; dietary advice to increase fiber intake]; and fiber dosage (low: <7.5 g/d; high: ≥7.5 g/d). Cut-off points for the variables used for group analysis were established according to Medical Subject Headings (MeSH) terms, previous publications [32], or the distribution of the obtained data. Moreover, to explore whether improvements in anthropometric measures may underlie the anti-inflammatory effects observed, we conducted a subgroup meta-analysis based on changes in BMI. For each inflammatory marker with sufficient data, studies were categorized into 2 subgroups: *1*) those in which the I group showed a statistically significant reduction in BMI, and *2*) those in which no significant BMI change was reported. Additionally, a sensitivity analysis excluding studies that reported significant between-group reductions in BMI during the I was performed.

The impact of each study on the specific outcomes was evaluated through a sensitivity analysis. Significant study outliers were identified through a study-by-study sensitivity analysis, where each study was sequentially omitted and the remaining data reassessed. Studies contributing over 30% to heterogeneity (based on changes to the I^2^ statistic) were excluded in the sensitivity analysis.

A random-effects meta-regression analysis was conducted to investigate potential sources of heterogeneity in the effect estimates, considering fiber quantity, type of fiber I, I duration, participants’ health condition, and baseline CRP as predictor variables. Covariates were selected based on their potential to influence the I effect and their availability across studies. Meta-regression was performed for inflammatory markers when at ≥10 studies were available [39].

The dataset of the meta-analysis was published on the open-access repository Zenodo and attributed the following digital object identifier, https://doi.org/10.5281/zenodo.14803906.

Publication bias was assessed by Egger’s linear regression test in meta-analyses including ≥10 studies, following the indications provided by the Cochrane Handbook for Systematic Reviews [39].

### Contact with authors

Attempts were made to contact the corresponding authors to request the full text when it was unavailable, verify eligibility, request outcomes by age group, or ask for additional information about outcome data for the meta-analysis (e.g., numeric values provided only in text or plots, unspecified units, parametric statistics).

### Ethical treatment of participants

All included studies reported approval by relevant ethics committees or institutional review boards and obtained informed consent from participants or their legal guardians, in accordance with ethical standards such as the Declaration of Helsinki.

## Results

### Study selection

Study identification and selection are detailed in the PRISMA flow chart (Supplemental Figure 1).

The search initially identified 2030 potentially relevant studies. Additionally, the manual search generated 5 records. However, 297 duplicates were identified and removed. Most articles (1520) were excluded by screening the title and abstract. After full-text screening, 26 studies fulfilled the inclusion criteria and were included in the qualitative synthesis of the systematic review, and 14 were included in the quantitative synthesis (meta-analysis).

### Description of the included studies

The 25 included primary studies analyzed a T of 1773 participants. Two included reports [44,45] presented different markers of inflammation from the same study and sample. Consequently, these articles were linked together and considered as 1 study, with all outcomes provided from both papers considered only once [39].

Characteristics of each study are presented in Table 1. Of the 25 studies included, 23 were parallel RCTs and 2 were crossover. These studies were published between 2006 and 2024. Among the included studies, 4 were conducted in Europe [44,[46], [47], [48]], 4 in Asia [[49], [50], [51], [52]], 4 in South and Central America [[53], [54], [55], [56]], 3 in Africa [[57], [58], [59]] and 10 in North America (of which 5 in the United States [[60], [61], [62], [63], [64]], 3 in Mexico [[65], [66], [67]] and 2 in Canada [68,69]).

**TABLE 1:** Characteristics and outcomes of studies included in the systematic review. Studies sorted by type of intervention (supplementation, fiber-rich product, advice).

Author Publication Year, Country Reference	I		Participants					Groups		Energy Intake Differences	Anthropometric differences	Outcomes
	Design	Duration (wk)	Sample size^1^	Age range Mean age	% Female	Health Condition	Weight status^2^ (BMI)	I - source and dose of fiber (g/d)	C - source and dose of placebo (g/d)			I–C differences for serum markers of CLGI
Feruś et al. 2018, Poland [44]	Single center, double-blind RCT	12	I: 16 C: 15	4–18 y (10 y)	I: 56.6 C: 63.4	Celiac disease	I: 17.1 C: 17.0	Oligofructose-enriched inulin (Synergy) 10 g/d	Maltodextrin 7 g/d	NR	BMI ↑ in I; no between-group differences	CRP: NS
Drabińska et al. 2019, Poland [45]	Single center, double-blind RCT	12	I: 16 C: 15	4–18 y (10 y)	I: 56.6 C: 63.4	Celiac disease	I: 17.1 C: 17.0	Oligofructose-enriched inulin (Synergy) 10 g/d	Maltodextrin 7 g/d	NR	NR	IL-1β: NS; IL-1ra: NS; IL-6: NS. IL-8: NS; IL-10: NS; IL-12p70: NS. TNF-α: NS
Nicolucci et al. 2017, Canada [68]	Single center, double-blind RCT	16	I: 20 C: 18	7–12 y (10.3 y)	I: 45.5 C: 40.0	Overweight or obesity	I: 26.3 C: 26.9	Oligofructose-enriched inulin 8 g/d	Maltodextrin 3.3 g/d	Isocaloric I	↓ Fat%, trunk fat, BMI *z*-score in I; between-group differences	CRP: NS; IL-1β: NS; IL-4: NS; IL-6: ↓∗; IFN-γ: NS; TNF-α: NS; IL-10: NS
Ho et al. 2019, Canada [69]	Single center, double-blind RCT	12	I: 17 C: 21	8–17 y (12.2 y)	I: 29.4 C: 66.7	T1D	NR	Oligofructose-enriched inulin 8 g/d	Maltodextrin 3.3 g/d	No changes	NR	IL-6: NS; IFN-γ: NS; TNF-α: NS IL-10: NS
Visuthranukul et al. 2022, Thailand [49]	Single center, double-blind RCT	24	I: 55 C: 55	7–15 y (10.4 y)	I: 45.4 C: 43.6	Obesity	I: 28.3 C: 28.5	Oligofructose-enriched inulin 13 g/d	Maltodextrin 11 g/d	Decrease within all groups; no between-group differences.	↓ BMI *z*-score and fat in all groups; no between-group difference	IL-1β: NS; IL-6: NS; TNF-α: NS
Paganini, Uyoga, Cercamondi et al. 2017, Kenya [57]	Single center, single-blind, RCT	3	I: 22 C: 28	6–14 mo (8.6 mo)	I: 54.6 C: 50.0	Iron deficiency	WLZ I: –0.47 C: –0.62	Fe + GOS 2.5 mg FeFum + 2.5 mg Fe NaFeEDTA + 7.5 g/d GOS + other components	Fe 2.5 mg FeFum + 2.5 mg Fe NaFeEDTA + other components	NR	NR	CRP: NS
Mikulic et al. 2024, Kenya [58]	RCT	3	I: 50 C: 59	6-11 mo (7.9 mo)	I: 52.8 C: 54.0	Iron deficiency	WLZ I: –0.60 C: –0.61 *z*-score	Fe + GOS/FOS 3.6 mg/d + 7.5 g/d	Fe (FeFum) 3.6 mg/d	NR	NR	CRP: NS
Paganini, Uyoga, Kortman et al. 2017, Kenya [59]	Single center, double-blind RCT	16	I: 52 C: 51	6.5–9.5 mo (7.4 mo)	I: 48.1 C: 45.1	Iron deficiency	NR	MNP sachet + GOS 7.5 g/d	MNP sachet + maltodextrin 10.5 g/d	NR	No differences	CRP: NS
Raes et al. 2010, Belgium [46]	Single center, double-blind RCT	26	I: 75 C: 81	Birth-26 wk	I: 43.0 C: 45.6	Healthy term infants	BW (g) I: 3510.7 C: 3340.3	GOS/FOS 0.6 g/100 mL/d	Infant formula	NR	NR	CRP: NS; IL-2: NS; IL-4: NS; IL-5 ↓∗; IL-10: NS; IFN-γ: NS; TNF-α: NS
Van den Berg et al. 2013, Netherlands [47]	RCT	3–30 d of life	I: 49 C: 53	(2.1 d)	I: 44.0 C: 38.0	Preterm infants	BW (g) I: 1320.0 C: 1230.0	GOS/FOS + pAOS enteral 80% + 20%	Maltodextrin	NR	NR	IL-1β: NS; IL-2: NS; IL-4: NS; IL-6: NS; IL-8: NS; IL-10: NS; IL-17: NS; IFN-γ: NS; TNF-α: NS;
Zheng et al. 2006, China [50]	Double-blind RCT	13–30 d	I: 32 C: 35	1–12 y (6.3 y)	C: 43.0 I: 34.0	Cancer (neuroblastoma, Wilms’ tumor, malignant teratoma, hepatoblastoma, and/or rhabdomyosarcoma)	WLZ I: 0.42 C: 0.70	Commercial enteral feed + FOS 400 mL/d + 2 g/L	Commercial enteral feed 400 mL/d	Macronutrient composition described; overall intake NR.	NR	IL-2: NS; IL-6: NS; TNF-α: NS
López-Velázquez et al. 2015, Mexico [65]	Double-blind RCT	8	I: 27 C: 31	20 + 7 d	NR	Healthy	NR	Metlin + metlos 0.5 g/100 mL of prebiotic (DF) 1–2 mo: 319.5–430.1 g/d 3 mo: 538–676 mg/d +probiotics	Probiotics	NR	NR	CRP ↓∗
Henao et al. 2018, Colombia [56]	Double-blind RCT	12	I: 60 C: 64	3–5 y (3 y)	NR	Healthy	NR	Yogurt enriched with β-glucans 350 mg/d	Yogurt 350 mg/d	NR	NR	IL-12: NS; IL-12p70: NS; IL-1β: NS; IL-6: NS; IL-10: NS; TNF-α: NS
González et al. 2021, Mexico [66]	Parallel, double-blind RCT	7	I: 50 C: 50	15–19 y (16 y)	I: 64.8 C: 60.7	Obesity	I: 31.8 C: 31	Plantago psyllium 10 g/d of DF	Rice flour 10 g/d	No differences between groups.	↓ BMI and waist in I; no significant between groups	IL-6 ↓∗
Fatahi et al. 2022, Iran [51]	Double-blind RCT	12	I: 31 C: 30	10–19 y (13 y)	T: 47.5	Overweight or obesity	I: 25.3 C: 24.7	Chitosan powder 3 g/d	Maltodextrin 3 g/d	Decrease in I group (200 kcal/d); no significant difference between groups.	↓ BMI, waist in I; significant between groups	Adiponectin ↑∗
Bseikri et al. 2018, United States [64]	Non-blinded RCT	8	I: 16 C: 15	14–18 y (15 y)	I: 35 C: 63	Asthma + Overweight or obesity	BMI (*z*-score): I: 2.14 C: 2.21	CHORI-bar 15 g/d of DF +other components (+8 exercise and nutrition classes)	8 exercise + nutrition classes	(↑ 260 kcal/d CHORI-bar) but no significant differences.	No differences	hs-CRP: NS Adiponectin: NS
Mietus-Snyder et al. 2020, United States [60]	RCT	8	I: 10 C: 8	14–18 y (15 y)	I: 75 C: 81	Obesity	I: 32.2 C: 35.9	Nutrient bar 18 g/d of DF +other components (+nutrition counseling and exercise)	Nutrition counseling and exercise	(↑ 220 kcal/d -bar) but no significant differences.	No differences	CRP: NS Adiponectin: NS
Anaya-Loyola 2020, Mexico [67]	Parallel, double-blind RCT	8	I: 33 C: 32	6–8 y	NR	Healthy	BMI (*z*-score): I: 0.78 C: 0.4	Mango by-product 1.08 g/d of DF +other components	Flavored water	No differences	No differences	CRP: NS; IL-1a ↑∗; IL-1b ↓∗; IL-2: NS; IL-4: NS; IL-6: NS; IL-8: NS; IL-10 ↑∗; TNF-α ↑∗; WBC: NS
Da Silva et al. 2020, Brasil [55]	Double-blind RCT	10	I: 8 C: 8	5–10 y (8 y)	I: 50 C: 50	Obesity	I: 23.54 C: 24.9	Chia seeds 3 g/d of DF (consumption of DF increased 3 g/d after I) +other components	Corn starch (consumption of DF decreased 0.6 g/d after C)	NR	No differences	hs-CRP ↓∗; IL-6 ↓∗; TNF-α ↓∗
Zambrana et al. 2021, Nicaragua [53]	Non-blinded RCT	24	I: 23 C: 24	(6 mo)	I: 47.82 C: 41.6	Healthy	Weight kg: I: 7.9 C: 8.1 Length cm: I: 66.2 C: 66.4	Rice bran Different doses by age: 6 mo: 0.25 g/d of DF 7–9 mo: 0.5 g/d 10 mo: 0.75 g/d 11 mo: 1 g/d 12 mo: 1.25 g/d +other components	-	NR	No differences	CRP: NS
Madsen et al. 2024, Denmark [48]	Crossover RCT	2 x 8	First period: I: 28 C: 27	8–13 y (11 y)	I: 16.57 C: 12.4	Overweight	BMI (*z*-score): T: 1.5 I: 1.5 C: 1.5	WG snacks 14–19 g/d of DF +other components	RG snacks	No differences	No differences	CRP: NS; IL-6: NS;
Vaz-Tostes et al. 2014, Brasil [54]	RCT	18	I: 41 C: 31	2–5 y (I: 47 mo; C: 42 mo)	I: 47 C: 46	Obesity	T: 16.32	Yacon flour preparations 0.14 g/d of DF (FOS)	-	No differences	↓ BMI in I; not significant between groups	IL-4: NS; IL-6: NS; IL-10: NS; TNF-α: NS;
Eisner et al. 2020, United States [61]	RCT	8	T: 38 I: - C: -	10–16 y	NR	Overweight or obesity	BMI percentile T, I, C: 92	Apples 8 g/d	Muffins	(↑ 240 kcal/d -bar) but no significant differences	No differences	CRP: NS Adiponectin: NS
Langkamp-Henken et al. 2012, United States [62]	RCT	6	I: 41 C: 42	11–15 y (12 y)	I: 44 C: 40	Healthy	% BMI category Healthy Weight: 55.5 Overweight: 14.5 Obesity: 30	Advice to consume WG-based foods (bread, cereals, or snacks) Consumption of DF increased 3 g/d after I	Advice to consume RG-based foods	No differences	NR	CRP: NS
Hajihashemi et al. 2014, Iran [52]	Crossover RCT	2 x 6	T: 44	8–15 y (11 y)	100	Overweight or obesity	T: 23.57 (≥85th percentile)	Advice to consume WG foods, brown rice, bran, etc. (0 g/d)	Asked not to consume any WG-phase foods.	No significant differences	No differences	CRP ↓∗
Hasson et al. 2012, United States [63]	RCT	16	I: 39 C: 30	14–18 y (15 y)	AA: 70.8 L: 48.1	Obesity	AA: 36 L: 33.9	Nutrition education program/advice focused on decreasing sugar intake and increasing fiber intake -	C	Nutrition (N) and Nutrition + Strength Training (N + ST) groups showed significant reductions in T energy intake (9.2% and 13.7%, respectively), compared to a 15.7% increase in C.	No differences	TNF-α: NS; IL-8: NS; Adiponectin: NS

Abbreviations: AA, African Americans; BMI, body mass index; C, control; CHORI-bar, name of the nutrient and fiber-dense bar; CLGI, chronic low-grade inflammation; CRP, C-reactive protein; DF, dietary fiber; FOS, fructooligosaccharides; GOS, galactooligosaccharides; hs-CRP, high-sensitivity C-reactive protein; I, intervention; IFN-γ, interferon-γ; IL, interleukin; L, Latinos; NR, not reported; NS, no statistically significant changes; RCT, randomized controlled trial; RG, refined grain; T, total; T1D, type 1 diabetes; TNF-α, tumor necrosis factor-α; WBC, white blood cell count; WG, whole grain; BW; FeFum, ferrous fumarate; MNP, micronutrient powder; NaFeEDTA, sodium iron ethylenediaminetetraacetate; pAOS, partially acid-oligosaccharide; WLZ, weight-for-length Z-score.

↑∗ The marker increased significantly by the fiber I (*P* < 0.05).

↓∗ The marker decreased significantly by the fiber I (*P* < 0.05).

1 Number of patients analyzed.

2 Baseline anthropometry: BMI when available, other measurements in the absence of BMI.

Regarding participants’ characteristics, 8 studies enrolled children and adolescents together [44,48,49,51,52,61,62,69]; 4, only adolescents [60,63,64,66], and 13 only children (7 of which studied infants [46,47,53,[57], [58], [59],^65^], 1 preschool children [54], and 5 school-age children according to MeSH definitions).

Participants in 4 studies had a chronic disease: cancer [50], type 1 diabetes mellitus [69], asthma [64], or celiac disease [44]. However, 21 studies investigated healthy subjects, 10 of which only included subjects with overweight or obesity, otherwise healthy. One study included healthy but preterm infants [47].

Supplemental Table 4 provides an overview of baseline serum inflammatory marker levels in pediatric populations included in the studies of the present systematic review. Initially, most studies reported biomarker levels within normal ranges, suggesting no underlying inflammation in the majority of cases. However, notable variability was observed, with some studies [54,69] reporting elevated or inconsistent levels of inflammatory markers such as IL-6 and TNF-α. CRP approached thresholds for low-grade inflammation (3 mg/L) in 2 studies [65,67].

The I of the majority of the studies (14) were with fiber supplementation, including: oligofructose-enriched inulin (4 studies), GOS and FOS combined or alone (6 studies), and 1 each with other fructans (1), β-glucans (1), psyllium (1), and chitosan powder (1). A T of 8 studies investigated the effects of items rich in fiber, including either products or foods used to supplement the diets of the participants, aiming to increase fiber intake (e.g., chia seeds, rice bran, bars, etc.). The remaining 3 studies provided food advice to increase fiber, WGs, or grain-based foods.

Fiber I doses ranged from 0.32 g/d to 25 g/d and treatment periods ranged from 3 wk to 6 mo, with a median and mode length of 8 wk.

Selected studies presented the following markers of inflammation. As pro-inflammatory markers, we retrieved information on: CRP, hs-CRP, IL-1α, IL-1β, IL-2, IL-2Ra, IL-5, IL-6, IL-8, IL-12, IL-18, TNF-α, white blood cells (WBC), platelets, and interferon-γ. The anti-inflammatory cytokines measured in selected studies were IL-4, IL-10, and adiponectin.

In addition to the commonly used markers for analyzing CLGI (CRP, IL-6, TNF-α, adiponectin, and WBC), data on other inflammatory markers were also retrieved. Despite being more specific to other immune responses (usually assessed in the context of acute inflammation and autoimmune conditions), these markers can provide valuable and supplementary insights into chronic inflammation.

In most articles, >1 inflammatory marker was assessed. Seven studies evaluated just the CRP as the only marker of inflammation [52,53,[57], [58], [59],^62^,^65^]. Van den Berg [47] only provides data about IL-1β, González et al. [66] about IL-6, and Fatahi et al. [51] about adiponectin. The trial that assessed the greatest number of inflammatory markers was Anaya-Loyola et al. [67], which combined 13 markers, followed by Nicolucci [68] (7), Raes et al. [46] (7), Drabińska [45] (5), and Henao [56] (5).

**FIGURE 1:**
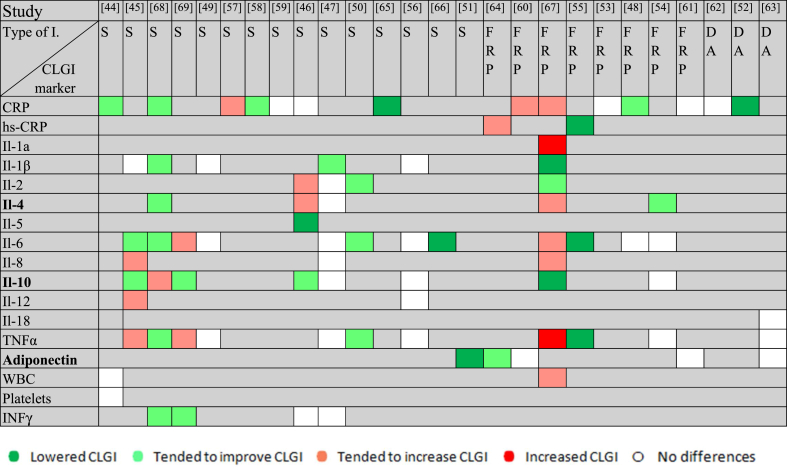
Effect of dietary fiber interventions (Is) on chronic low-grade inflammation (**CLGI**) markers. This figure shows the effect of the dietary fiber Is of each study on CLGI. When after the I the pro-inflammatory markers decreased or the anti-inflammatory markers increased compared to placebo, green color was assigned, indicating an improvement of CLGI. If this change was not statistically significant, a lighter green color was assigned. When after the I pro-inflammatory markers increased or the anti-inflammatory markers decreased compared to placebo, red color was assigned, indicating an increase in CLGI. If this change was not statistically significant, a lighter red color was assigned. No changes in inflammatory markers are shown in white. **CRP**, C-Reactive Protein; DA, dietary advice to increase fiber intake; FRP, fiber-rich product; **hs-CRP**, high-sensitivity C-reactive protein; **IFN-γ**, interferon-γ; **IL,** interleukin; S, supplementation with dietary fiber; **T1D**, type 1 diabetes; **TNF-α**, tumor necrosis factor-α; **WBC**, white blood cell.

The outcomes of the different markers in the same trial were not always consistent (didn’t change in the same sense/direction), as shown in Table 1 [[44], [45], [46], [47], [48], [49], [50], [51], [52], [53], [54], [55], [56], [57], [58], [59], [60], [61], [62], [63], [64], [65], [66], [67], [68], [69]] and Figure 1.

### Fiber consumption and CLGI

**FIGURE 2:**
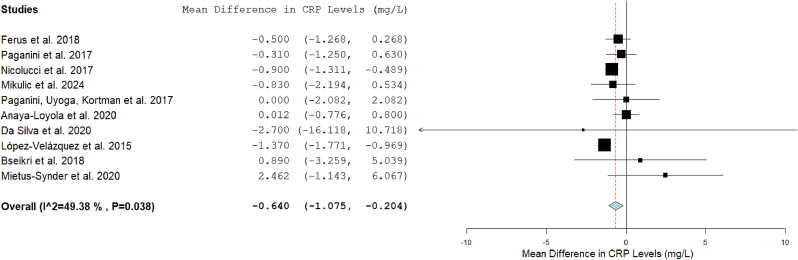
Forest plot of the effects of dietary fiber vs control on CRP.

**FIGURE 3:**
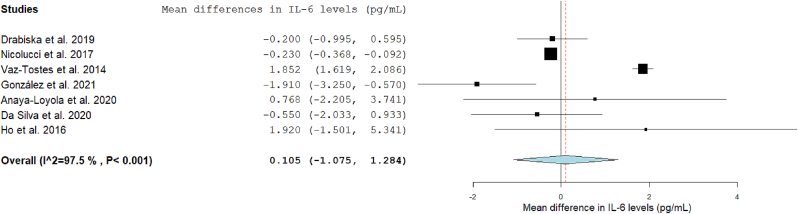
Forest plot of the effects of dietary fiber vs placebo on IL-6.

**FIGURE 4:**
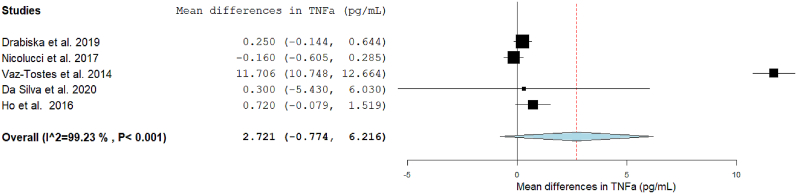
Forest plot of the effects of dietary fiber vs placebo on TNFα.

**FIGURE 5:**
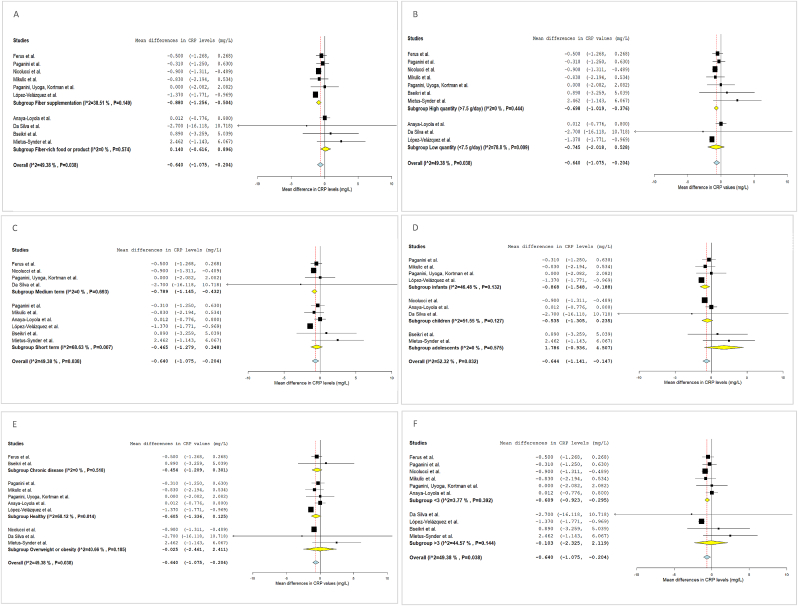
Subgroup analysis of the effects of dietary fiber on CRP levels.

**FIGURE 6:**
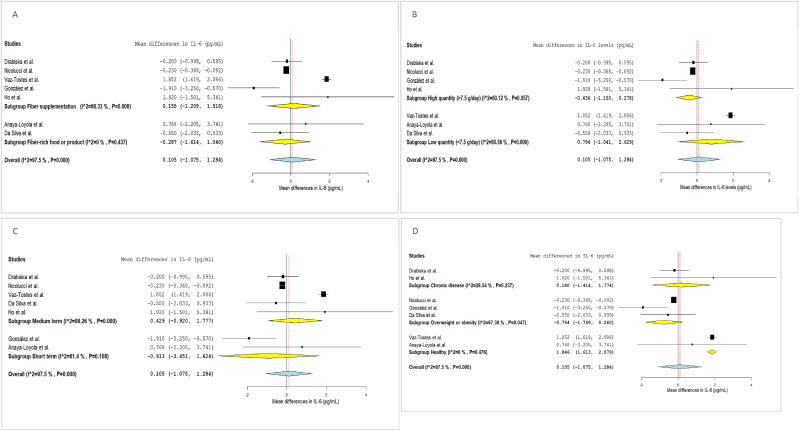
Subgroup analysis of the effects of dietary fiber on IL-6 levels.

**FIGURE 7:**
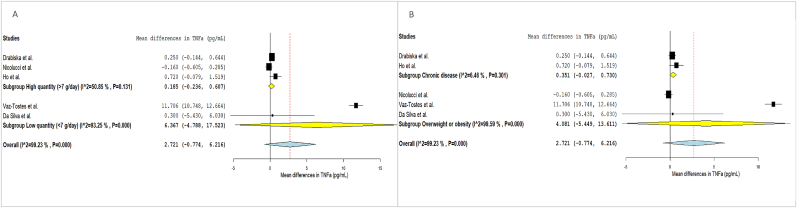
Subgroup analysis of the effects of dietary fiber on TNFα levels.

The outcomes of the systematic review and of each meta-analysis are reported in Table 1, FIGURE 1, FIGURE 2, FIGURE 3, FIGURE 4. Results from subgroup analyses performed are included in FIGURE 5, FIGURE 6, FIGURE 7.

#### DF and CRP

CRP concentrations were reported in a T of 17 included studies. Three studies expressed the outcome as hs-CRP.

In 6 out of the 17 studies reporting CRP changes, CRP decreased but not significantly [44,48,57,58,65,68]; in 3, the concentration increased also not significantly [60,64,67]; in 6 there were no differences between I and C groups [46,53,56,59,61,62] and in 2 trials there was a significant reduction in hs-CRP concentration after the I with fiber, compared to the comparison group [52,55].

Because they had complete information on CRP mean changes after fiber I for the experimental and C groups, 10 eligible studies were included in the meta-analysis, that showed that CRP concentrations decreased significantly by the DF I compared to the C (MD: –0.640; 95% CI: –1.075, –0.204) (Figure 2). Heterogeneity among studies for CRP was moderate (I^2^: 49%).

Subgroup analysis (Figure 5) of fiber types used in each I showed that fiber supplementation (oligofructose-enriched inulin, GOS, GOS and FOS, and fructans) led to a greater decrease of serum CRP concentration compared to Cs (MD: –0.880; 95% CI: –1.256, –0.504; I^2^: 38%), whereas Is with fiber-rich foods or products led to no significant differences in CRP concentrations (MD: 0.140; 95% CI: –0.616, 0.896; I^2^: 0%).

With regard to subgroup analysis by quantity of fiber, a significant reduction in CRP was only observed in the studies in which the T fiber intake was 7.5 g/d higher in the I group than in the C group (MD: –0.698; 95% CI: –1.019, –0.376; I^2^: 0%). In those participants who consumed <7.5 g/d of fiber, more than those in the C group (that, in fact, was <3 g in all studies included), a reduction in CRP was observed, although this was not statistically significant (MD: –0.745; 95% CI: –2.018, 0.528; I^2^:79%). Heterogeneity in studies with low quantities of fiber was high.

Additionally, the effect was stronger in medium-term Is (10–18 wk) (MD: –0.789; 95% CI: –1.145, –0.432; I^2^: 0%) than in short-term Is, in which it was not significantly different (MD: –0.465; 95% CI: –1.279, 0.348; I^2^: 68%).

Regarding subjects’ age, the effect was statistically significant in infants (MD: –0.868; 95% CI: –1.548, –0.188; I^2^: 46%) but not in children (MD: –0.535; 95% CI: –1.305, 0.235; I^2^: 52%) nor in adolescents (MD: 1.786; 95% CI: –0.936, 4.507; I^2^: 0%). Some studies included participants from different age groups, so they weren’t included in this subgroup analysis [44,61].

In terms of health condition, there were no significant differences in CRP after the Is in healthy participants (MD: –0.605; 95% CI: –1.336, 0.125; I^2^: 68%), in those with overweight or obesity (MD: –0.025; 95% CI: –2.461, 2.411; I^2^: 41%) nor in those with chronic diseases (MD: –0.454; 95% CI: –1.209, 0.301; I^2^: 0%).

Furthermore, CRP reductions were significant in participants with lower baseline CRP (<3 mg/L) (MD: –0.609; 95% CI: –0.923, –0.295; I^2^: 4%).

Sensitivity analysis (Supplemental Figure 2) after excluding 1 study each time was performed, and the results obtained were similar, both in direction and magnitude of effect and statistical significance, in most of the attempts. This indicates that the analysis is robust. Only after excluding Nicolucci et al. [68] study, the reduction in CRP was no longer significant, and if we excluded Anaya-Loyola et al. [67] study, the magnitude of the effect was bigger.

The meta-regression results indicate that the type of fiber I is a significant moderator of CRP changes (Supplemental Table 5). Specifically, Is with fiber supplementation were associated with significantly greater reductions in CRP compared to those using fiber-rich products (β: –1.358, 95% CI: –2.396, –0.320; *P* = 0.010). Other factors, including fiber quantity, I duration, baseline CRP concentrations, and health conditions, did not show statistically significant effects on inflammatory outcomes. However, the omnibus *P* value (*P* = 0.009) indicates that the overall model is statistically meaningful, suggesting that the collective influence of the included variables provides insights into the relationship between fiber Is and inflammation. Although individual factors such as I duration or health condition did not independently impact CRP concentrations, the type of fiber I, particularly through fiber supplementation, emerged as a significant determinant.

Seven studies were not included in the meta-analysis as they did not meet the inclusion criteria: 3 studies reported the values of the marker only at the end of the I [46,50,53], another was a crossover RCT and did not provide baseline values after the washout period [48]. Two studies differed significantly from the others in terms of I, as it was based on advice [62], and in 1 of them, no greater amount of fiber was consumed in the I group compared to the C group as a result of the dietary counseling I [52]. Another study did not clarify the number of subjects in each group [61].

In 4 studies not included in the meta-analysis, different kinds of Is with fiber (prebiotics GOS + FOS [46], WGs [48], rice bran [53], apple [61], and advice of increasing fiber intake [62]) did not lead to a significant difference in serum CRP compared with placebo. Another trial not included in the meta-analysis, which was based on advice to increase DF, showed a significant reduction in hs-CRP concentration after the I, compared to C [52].

#### DF and IL-6

IL-6 was measured in 11 eligible studies, 7 of which reported the data in a form suitable for inclusion in the meta-analysis (mean change in IL-6 concentration following fiber I). The analysis showed no significant differences in IL-6 concentrations after the DF I compared to the C (MD: 0.105; 95% CI: –1.075, 1.284) (Figure 3). Heterogeneity among studies for IL-6 was high (I^2^: 97.5%).

Figure 6 presents the subgroup analysis. Changes in this serum marker remained nonsignificant across subgroups defined by type, quantity of fiber, or duration of the I. In terms of health condition, the subgroup analysis showed that in healthy subjects, IL-6 concentrations were higher following fiber I (MD: 1.846; 95% CI: 1.613, 2.079; I^2^: 0%); no significant differences were observed in the other 2 groups. Subgroup analysis by age was not feasible due to the limited number of trials: only 1 trial focused on adolescents, 2 studies included participants from mixed age groups, and the remaining trials were conducted exclusively in children.

A sensitivity analysis was performed, and the changes remained statistically nonsignificant in all analyses (Supplemental Figure 3). Vaz-Tostes et al. [54] study contributed to over 50% heterogeneity (based on changes to the I^2^ statistic). Thus, it was removed from the analysis in the sensitivity analysis, resulting in a change of the effect’s direction. Although still not significant, the Is with fiber in the remaining studies led to a reduction in IL-6 concentrations (MD: –0.379; 95% CI: –0.917, 0.158; I^2^: 38.5%).

Four studies were not included in the meta-analysis as 2 of them did not provide numeric information on changes in inflammatory markers [49,56], and another was a crossover RCT and did not report baseline values after the washout period [48]. The last trial was not included, as the reported concentrations differed widely from those observed in the other included trials [50].

In these studies not included in the meta-analysis, Is with fiber supplements of prebiotics did not lead to significant differences in serum IL-6 compared with placebo.

#### DF and TNF-α

TNF-α was analyzed in 11 eligible studies, 5 of which reported the data in a format suitable for inclusion in the meta-analysis (mean change in TNF-α concentration following fiber I). DF Is did not have a significant effect on TNF-α compared to the C group (MD: 2.721; 95% CI: –0.774, 6.216), albeit with high heterogeneity (I^2^: 99.23%) (Figure 4).

Subgroup analyses by age and type of fiber were not feasible, as 2 trials included both children and adolescents together, and the remaining focused solely on children. Additionally, only 1 I was based on fiber-rich products, whereas the others used supplemented fiber. All studies involved medium-term Is. Subgroup analysis by quantity of fiber and participants’ health condition showed no significant differences between groups (Figure 7).

A sensitivity analysis was conducted (Supplemental Figure 4), and the effect remained similar after excluding each article except when the article of Vaz-Tostes et al. [54], identified as an outlier, was removed. This exclusion resulted in a smaller increase in TNF-α, still not significant (MD: 0.17; 95% CI: –0.19, 0.53). The Vaz-Tostes et al. [54] study contributed to >70% of the heterogeneity (I^2^), which was reduced to 26% after its removal.

Six studies were not included in the meta-analysis as 2 of them reported no numerical results, only presenting data in plots and text [49,56], and 2 others showed extreme values that differed widely from the rest of the included trials, possibly due to the use of a different analysis kit [50,67]. The study by Raes et al. [46] only reported marker concentrations after the I, and Hasson et al. [63] was the only trial to investigate an I based on advice in this meta-analysis. None of these trials found significant differences in TNF-α after the DF I.

#### DF and other markers of CLGI

A meta-analysis was not possible for the remaining biomarkers (IL-1α, IL-1β, IL-2, IL-4, IL-5, IL-8, IL-10, IL-12, IL-18, adiponectin, WBC, platelets, and INF-γ) due to the lack of studies reporting outcomes in a metric suitable for inclusion.

Results of the systematic review are presented in Table 1. Adiponectin was analyzed in 5 studies. It increased in 2 trials after the Is with DF: 1 with a product that provided 15 g of additional fiber per day [64] and another after consuming a supplement of 3 g of chitosan powder per day [51]. However, adiponectin did not change significantly after Is based on products providing <10 g of fiber [60,61] or on advice to increase fiber intake [63]. In the Hasson et al. [63] trial, adiponectin responses varied by race, increasing for Latinos and decreasing for African Americans, although these changes were not statistically significant.

Seven trials investigated IL-10 following DF Is. Three reported no significant differences [54,56,68]. In the other 3 trials, this anti-inflammatory cytokine increased but not significantly [45,46,69], and in 1 trial IL-10 was significantly higher after an I with a fiber-rich product [67].

Cytokine IL-1β was analyzed in 6 included articles. In 3 of them, there were no significant differences after supplementation with different prebiotics [45,49,56], and in 2 studies with similar Is there were trends toward lower levels, still not significant [47,68]. One study found a significant decrease in IL-1β in the I group compared to placebo following the consumption of a fiber-rich product [67].

No significant differences were observed for any other marker of inflammation among the included studies, as summarized in Table 1 and Figure 1. WBC [44,46,67] and INF-γ [46,68,69] were analyzed in 3 trials each 1, and none of the Is (with supplements of fiber or with fiber-rich products) led to significant changes. Similarly, no differences were found for IL-4 [46,54,67,68], IL-8 [45,63,67], IL-2 [46,50,67], platelets [44], IL-12 [56], or IL-18 [63].

However, a significant increase in IL-1α was observed after an I with a mango product [67], and IL-5 concentrations decreased significantly following supplementation with GOS and FOS [46].

### Assessment of energy intake and BMI changes as potential confounders

Among the 26 studies included in the systematic review, only 3 (Fatahi et al. [51], 2022; Hasson et al. [63], 2012; Visuthranukul et al. [49], 2022) reported a statistically significant decrease in energy intake within ≥1 I group. However, only Hasson et al. [63] (2012) reported a statistically significant reduction in energy intake when compared to the C group, indicating a between-group effect. In contrast, Fatahi et al. [51] and Visuthranukul et al. [49] observed reductions within groups, but these did not reach statistical significance in comparison to Cs. None of these studies were included in the meta-analysis of energy intake because they did not meet the eligibility criteria. The remaining studies either did not report changes in energy intake or explicitly stated that energy intake remained unchanged. Thus, in most included trials, the observed clinical effects occurred independently of any energy restriction.

To explore whether improvements in anthropometry may underlie the anti-inflammatory effects observed, anthropometric outcomes across studies were examined (Table 1). Reporting was inconsistent, and only 2 trials [51,68] reported significant reductions in obesity-related measures alongside significant between-group differences favoring the fiber I. Fatahi et al. [51] observed reductions in BMI and waist circumference with increased adiponectin concentrations. Nicolucci et al. [68] reported concurrent reductions in *z*-score and body fat percentage along with lower IL-6 concentrations. Other studies, such as González et al. [66], reported within-group BMI reductions without significant between-group differences, yet still observed improvements in inflammatory markers.

Exploratory sensitivity meta-analyses were conducted for CRP, IL-6, and TNF-α, excluding studies reporting significant between-group reductions in BMI (Nicolucci et al. [68]). For CRP (*n* = 6), the effect remained significant (MD: –1.546; 95% CI: –2.993, –0.098). For IL-6 (*n* = 6) and TNF-α (*n* = 4), results remained nonsignificant (IL-6 MD: 0.193; 95% CI: –1.317, 1.703; TNF-α MD: 3.407; 95% CI: –2.084, 8.897).

### Quality assessment

The quality of each study is available in Supplemental Table 6. Quality scores for the studies included ranged from 4 to 10 points out of a T of 10, indicating moderate and high quality. Most (92.3%) of them were classified as high-quality studies. The mean quality score was 7.8. All included studies met the criterion of external validity.

### Risk of bias assessment

Four studies were found to have a low risk of bias, 7 had a moderate risk, and 14 were identified as having a high risk of bias, mostly due to some concerns found in the second and fifth domains, corresponding to deviations from intended I and bias in selection of the reported result. Risk of Bias Visualization (RobVis) plots are available in supplementary materials (Supplementary Figure 5).

### Publication bias

The results of Egger’s test indicated a significant intercept (*z* = 2.45, *P* = 0.014), suggesting the presence of publication bias. Specifically, the asymmetry in the funnel plot suggests that smaller studies might be overestimating the I effect.

## Discussion

To the best of our knowledge, this is the first systematic review to perform a quantitative systematic analysis using exclusively RCTs to evaluate the effect of DF intake on inflammatory markers in children and adolescents. The results of this review show considerable heterogeneity, reflecting inconsistencies in the effects of fiber intake on different inflammatory markers, as well as among different fibers Is targeting the same marker. However, the results of the MA indicated a significant reduction in CRP concentrations in response to DF Is, whereas other inflammatory markers such as IL-6 and TNF-α did not show significant changes. Our findings underline the complexity of fiber’s impact on inflammation and the need for further research to explore the underlying mechanisms. Notably, baseline inflammatory marker concentrations were within the normal ranges in most of the studies included in this review, which may have limited the ability to detect significant changes in response to the Is.

In terms of circulating CRP, the most widely studied inflammatory marker, the results from the MA that included 10 studies with children and adolescents reveal that DF Is may have a beneficial effect on reducing inflammation in the pediatric population. When comparing the present study with similar studies, a recent systematic review [70], which primarily included observational studies, assessed the associations between various dietary components and markers of inflammation in children and adolescents; and regarding fiber, 6 of the included studies of which only 1 was an I trial found no association between fiber consumption and CRP, whereas 2 reported an inverse association. In contrast, the present study focuses exclusively on I studies, and, additionally, provides a quantitative synthesis through a meta-analysis, which reflected a decrease in CRP following fiber consumption. This methodological approach not only complements the existing evidence but also provides more robust conclusions about the impact of DF on this inflammation marker.

Another review conducted in adults with overweight or obesity, but that performed a meta-analysis, showed similar effects of DF intake on circulating CRP concentrations, showing a significant reduction in this inflammatory marker compared to the C group (–0.37 mg/L; (95% CI: –0.74, 0 mg/L) [32]. Similar to our study, Jiao [32] included Is based on different fiber sources or strategies to increase DF, including fiber supplementation (e.g., with psyllium) and fiber-rich foods (e.g., WGs), considering them together in the meta-analysis.

Subgroup analysis showed a greater significant reduction in CRP when the type of I was DF supplementation, or the amount of fiber was higher than 7.5 g/d, or the I was medium-term (10–18 wk), and the population group consisted of infants. Furthermore, there was a significant reduction in CRP concentrations among studies with baseline CRP < 3 mg/L compared to those with higher baseline inflammation.

The results of the meta-regression for CRP changes align with those from the subgroup analysis, both indicating that fiber supplementation was more effective than fiber-rich foods in reducing CRP concentrations. Specifically, the meta-regression, adjusting for covariates such as fiber dose and I duration, supported that those Is using fiber supplementation resulted in significantly greater reductions in this inflammatory marker. However, although the subgroup analysis suggested statistically significant effects for longer-duration Is, lower baseline CRP, and specific populations, such as infants, these effects were not significant in the meta-regression after adjusting for confounders. This highlights the limitations of subgroup analysis, which does not account for the simultaneous influence of multiple factors. Together, these approaches provide evidence that the mode of fiber delivery may be an important factor in its anti-inflammatory effects. The biological significance of the findings should be interpreted with caution, as the observed reduction in CRP concentrations, although statistically significant, was moderate. Clinically, serum CRP concentrations are used to classify cardiovascular disease risk into low (<1 mg/L), intermediate (1–3 mg/L), and high (>3 mg/L) [71]. Even small reductions in CRP concentrations may be relevant for individuals at higher risk [72,73]. Nevertheless, none of the included studies reported the effect size using Cohen’s d, making it difficult to assess the magnitude of the I’s impact. Taking into account all the above, further research is needed to determine optimal dosages and types of DF, as well as to identify target populations and understand the mechanisms by which fiber influences the immune system and inflammatory molecules in the prevention of chronic diseases.

Regarding other markers of CLGI, such as TNF-α and IL-6, MA, and most of the studies included in this systematic review did not report significant changes following the Is. The lack of significant effects for TNF-α and IL-6 in the MA may be partly explained by the smaller number of included studies and the greater variability in effect estimates compared to CRP. In addition, the different kinetics of these markers may contribute to this result, as cytokines like IL-6 and TNF-α peak rapidly and transiently, whereas CRP shows a more stable and sustained response, potentially making it more suitable for detecting changes in CLGI [74,75].

Due to the lack of similar evidence in pediatric populations, we compared our findings with those from other age groups. For instance, a pilot RCT involving pregnant females found no significant difference in IL-6 concentrations following supplementation with GOS compared to a C group that received a different type of fiber, FOS, albeit at lower concentrations [76]. In the elderly, GOS supplementation led to decreased concentrations of IL-6 and TNF-α [77]. Additionally, an RCT found lower serum TNF-α concentrations in adults with chronic dermatological diseases after fiber supplementation, specifically with xylorhamnoglucuronan [78]. In contrast, Anaya-Loyola et al. [67] study reported increased TNF-α concentrations after an I with mango by-products that contained a small amount of fiber alongside many other components. It is important to note that isolating the effect of fiber in such cases, where Is are based on products containing multiple constituents, is particularly challenging.

In the present review, most studies examining other inflammatory biomarkers, such as IL-1β and IL-10, did not find significant differences. Only 1 study reported a decrease in the pro-inflammatory marker IL-1β and an increase in the anti-inflammatory marker IL-10 [67].

Most of the studies measuring adiponectin concentrations showed an increase following fiber Is. A similar outcome was observed in the RCT by Hume et al. [79], where children with overweight and obesity consumed 8 g of oligofructose-enriched inulin per day, for 16 wk. This I not only resulted in significantly higher feelings of fullness but also led to increased fasting adiponectin concentrations compared to the placebo [79].

These results suggest that although DF may have some potential to influence the markers outlined above, the evidence remains inconsistent, and further research is needed to clarify its role in modulating anti-inflammatory responses. Moreover, most of the studies that discussed our findings were conducted in adults, highlighting the need for further research in the pediatric and adolescent population.

Our findings do not allow us to conclusively determine whether the effects of DF are mediated through changes in anthropometry and reductions in adiposity. This limitation arises from the fact that many of the included RCTs did not report sufficient information on changes in BMI or body composition. Among those that did, only 2 studies [51,68] showed concurrent reductions in both adiposity and inflammatory markers, which could lend some support to the hypothesis. However, the overall body of evidence does not allow for a firm conclusion due to the limited and inconsistent reporting of anthropometric outcomes.

Furthermore, our exploratory meta-regression and subgroup analyses did not reveal a robust association between participants’ BMI and changes in inflammatory markers. Similarly, sensitivity analyses excluding studies with significant BMI reductions in the I group [68] yielded comparable results. These findings underscore the importance of future trials systematically assessing and reporting both inflammatory and anthropometric outcomes to better elucidate potential mediating pathways.

The mechanisms through which DF influences inflammation likely involve its effects on gut microbiota, which plays a central role in immune modulation. These Is lead to an increased abundance of beneficial species such as Bifidobacterium and Lactobacillus spp., which play a crucial role in maintaining intestinal barrier integrity and balanced gut permeability, thus preventing the diffusion of bacterial antigens that could trigger chronic inflammation [80,81]. Additionally, short-chain fatty acids produced by gut bacteria further inhibit the production of pro-inflammatory cytokines [82,83].

The promotion of healthy eating habits is strategically important among children and adolescents, given the critical phase of growth and development and the benefits generated for health in the short and long-term. Although in this study, greater reductions in CRP were observed in Is involving fiber supplementation and couldn’t be assessed in dietary counseling in the subgroup analyses, the critical role of overall diet quality and nutritional counseling strategies in promoting healthy eating and preventing noncommunicable diseases is undeniable [84,85]. Along these lines, the latest dietary guidelines unanimously recommend and emphasize the importance of including fiber in the diet from a variety of food sources and fiber-rich products such as WGs, vegetables, fruits, and pulses [86,87]. As important as the type and source of DF is the quantity ingested. Nutritional guidelines advise 14 g of fiber per 1000 kcal from early childhood, recommending 15 g for children aged 2–5 and increasing to 21–25 g for older children and teens [24,25]. The United States Food and Drug Administration aligns with these recommendations, suggesting 28 g/2000 kcal for those aged 4 and up [26]. Despite these guidelines, actual DF consumption remains below recommendations for this age group. Less than 3% of all age and sex subgroups of children and adolescents have adequate habitual DF intake [31]. In European and North American children, the mean daily intake of DF is ∼14.8 g for all ages and ethnicities [[28], [29], [30], [31]].

The variability in the response of inflammatory markers to I between studies and within the same study can be explained by considering some factors. Among them, individual variability, including genetic, ethnic, and baseline health status differences, could influence the inflammatory response. Furthermore, inflammatory markers are regulated by complex and diverse mechanisms, which can result in discordant responses. In addition, inflammation has acute and chronic phases, and some markers may respond to I at different times. In parallel, the short timing of post-I measurements in some studies could compromise the observation of more significant results.

The present study has some limitations. A key limitation is that some included studies were conducted in infants, children, or adolescents with specific health conditions, such as obesity, iron deficiency, or celiac disease, which may limit the generalizability of the findings to the broader healthy pediatric population. To address this, we performed subgroup analyses by health status (i.e., healthy participants; those with overweight or obesity; and those with chronic inflammatory conditions such as asthma, cancer, celiac disease, or type 1 diabetes; Figure 5E). Although a trend toward reduced CRP concentrations was observed in studies with healthy participants and those with chronic conditions, subgroup effects were no longer statistically significant.

Secondly, the heterogeneity among the included studies may impact the consistency of the results and make it impossible to carry out meta-analysis for other inflammatory markers. Moderate heterogeneity observed among the studies on CRP was partly explained by subgroup analyses. Heterogeneity among studies on TNF-α and IL-6 was very high and was partly attributed to participants’ health condition or the quantity of fiber consumed during the I. Heterogeneity in these 2 markers was principally due to the study by Vaz-Tostes et al. [54], which involved very low fiber doses. Sensitivity analysis revealed that excluding this study reduced heterogeneity and shifted the effect of the I on TNF-α and IL-6. Nevertheless, heterogeneity was not fully explained for any of the 3 markers analyzed using the methods described. It is thought that other potential sources of heterogeneity could include the number of participants (*N*) in the studies or their ethnicity. To further address this limitation, it is noteworthy that a meta-regression was conducted where methodologically possible.

Another limitation of the present study was the variation in study quality, particularly regarding the item “deviations from intended Is.” Nevertheless, all the studies included in the meta-analysis were of high quality (excellent or good), except for 1 study, which had moderate methodological quality and was included in the TNF-α and IL-6 meta-analyses. The study by Vaz-Tostes et al. [54], having low quality, was the 1 excluded in the sensitivity analysis.

It is also worth mentioning that 3 articles in the review were conducted by the same group of researchers, led by Zimmermann, and involved the same population [[57], [58], [59]]. Although we cannot guarantee complete independence of the samples, we ensured the studies were considered independent by verifying trial registration numbers, dates, and participants’ ages. This methodological rigor supports the reliability of our findings.

Lastly, publication bias was detected through Egger’s test, suggesting potential overestimation of results due to unpublished studies with nonsignificant outcomes. It is also possible that the I does not lack effect, but rather that there is a lack of studies showing negative or null effects. To address this bias in future research, we recommend the inclusion of unpublished studies and the use of clinical trial registries to obtain a more comprehensive evidence base.

As strengths, this study is the first to perform a quantitative systematic analysis using exclusively RCTs to evaluate the effect of DF intake on inflammatory markers, assessing CLGI, in children and adolescents. Additionally, this study explored various types, durations, and amounts of fiber in the Is, potentially indicating more effective strategies for promoting healthy eating habits and reducing CLGI in the pediatric population. This approach provides a clearer understanding of how different factors may influence the inflammatory response and helps identify which populations might benefit most from DF.

Although most studies have focused on CRP as an inflammatory marker, this study advances the field by including meta-analyses of other inflammatory markers, thereby expanding the understanding of DF’s impact on the inflammatory process.

Another strength of this review is the detailed evaluation of energy intake across included studies. Although fiber intake is often associated with satiety and potential reductions in energy consumption, only a small subset of studies in our analysis reported a decrease in energy intake during the I. Notably, in 2 of the studies where a reduction was observed [49,51], the decrease was not statistically significant between groups. Moreover, several Is added calories (e.g., fiber bars, fortified cereals), suggesting that the beneficial outcomes observed, particularly in inflammatory markers, are unlikely to be attributable to energy restriction alone. This reinforces the hypothesis that DF may exert direct physiological effects on immune function independent of energy intake.

In conclusion, the findings of this systematic review and meta-analysis indicate that DF intake may impact inflammatory markers in children and adolescents. The most consistent effect was observed for CRP, where meta-analysis and meta-regression were feasible and reveale significant reductions in this inflammatory marker, especially in intervention based on fiber supplementation. However, changes in other key inflammatory markers, such as IL-6 and adiponectin, are less consistent, with some studies reporting improvements whereas others find no significant changes. Other inflammatory markers assessed show either contradictory results or no changes. The findings support the need for further studies, given that DF consumption may represent a potential strategy to modulate certain inflammatory markers and reduce the risk of CLGI from early stages of life.

## Author contributions

The authors’ responsibilities were as follows– MIB-T, AMS-P, LAM: designed research; MIB-T, AFS: conducted research, analyzed data, and wrote paper; MIB-T, MLM-B: performed statistical analysis; IR-DT, MLM-B, AMS-P, LAM: critically revised the manuscript for intellectual content; MIB-T, AMS-P, LAM: had primary responsibility for final content; and all authors: read and approved the final manuscript.

## Data availability

Data described in the manuscript, code book, and analytic code will be made publicly and freely available without restriction at https://doi.org/10.5281/zenodo.14803906.

## Funding and aknowledgements

The paper is based on a scientific session at the 10th International Conference on Nutrition and Growth, presented at the Symposium ‘The quality of diet: do we really have a science-based data bank?’ during the Nutrition & Growth Conference (London, 30 March 2023), supported by Soremartec Italia S.R.L. Its contents are solely the responsibility of the authors.

This work was supported by a predoctoral research grant from the Aragón Regional Government (Diputación General de Aragón).

## Conflict of interest

The authors report no conflicts of interest.
