# Supplementary material for: Effect of Dietary Fiber Intake on Chronic Low-Grade Inflammation in Children and Adolescents: A Systematic Review and Meta-analysis of Randomized Controlled Trials
Source: Curr Dev Nutr. 2025 Jul 24;9(9):107511. doi: 10.1016/j.cdnut.2025.107511 (PMC12423409; doi:10.1016/j.cdnut.2025.107511)
Supplement: multimedia component 1 [file mmc1.docx]

**Supplementary Materials (intended for publication)**

**Supplementary Table 1**

|  | **WEB OF SCIENCE** | **SCOPUS** | **PUBMED** |
| --- | --- | --- | --- |
| Fiber keywords | ((((((((((((((((((((((((((((((((((((((((((((((((TS=(fibre*)) OR TS=(fiber*)) OR TS=(roughage)) OR TS=(lignin)) OR TS=(chitin*)) OR TS=(phytate*)) OR TS=("resistant starch")) OR TS=(dextrin*)) OR TS=(RS1)) OR TS=(RS2)) OR TS=(RS3)) OR TS=("resistant oligosaccharide*")) OR TS=("α-Galactoside*")) OR TS=(β-fructo-oligosaccharide*)) OR TS=(FOS*)) OR TS=(α-galacto-oligosaccharide*)) OR TS=(GOS*)) OR TS=(β-galacto-oligosaccharide*)) OR TS=(TOS*)) OR TS=(xylo-oligosaccharide*)) OR TS=(XOS*)) OR TS=(arabino-xylo-oligosaccharide*)) OR TS=(AXOS*)) OR TS=(Polydextrose)) OR TS=("Non-starch polysaccharide*")) OR TS=(cellulose)) OR TS=(Hemicellulose*)) OR TS=(Glucuronoxylan*)) OR TS=(Heteroxylan*)) OR TS=(x?loglucan*)) OR TS=(mannan*)) OR TS=(galactomannan*)) OR TS=(glucomannan*)) OR TS=(galactoglucomannan*)) OR TS=(pectin*)) OR TS=(Polygalacturonic)) OR TS=(gum*)) OR TS=(mucilage*)) OR TS=(Arabinogalactan)) OR TS=(xanthan)) OR TS=(alginate*)) OR TS=(agar)) OR TS=(agar-agar)) OR TS=(carrageenan)) OR TS=(inulin)) OR TS=(fructan*)) OR TS=(β-glucan*)) AND ((((((TS=(consum*)) OR TS=(eat*)) OR TS=(diet*)) OR TS=(nutri*)) OR TS=(food*)) OR TS=(intake))OR TS=(supplement*)) OR (((TS=(“dietary fiber*”)) OR TS=(“dietary fibre*”)) OR TS=(“alimentary fiber*”)) OR TS=(“alimentary fibre*”) | ((((TITLE-ABS-KEY ("dietary fiber*") OR TITLE-ABS-KEY ("dietary fibre*") OR TITLE-ABS-KEY ("alimentary fiber*") OR TITLE-ABS-KEY ("alimentary fibre*")) OR ((TITLE-ABS-KEY (consum*) OR TITLE-ABS-KEY (eat*) OR TITLE-ABS-KEY (diet*) OR TITLE-ABS-KEY (nutri*) OR TITLE-ABS-KEY (food*) OR TITLE-ABS-KEY (intake) OR TITLE-ABS-KEY (supplement*)) AND (TITLE-ABS-KEY (fibre*) OR TITLE-ABS-KEY (fiber*) OR TITLE-ABS-KEY (roughage) OR TITLE-ABS-KEY (lignin) OR TITLE-ABS-KEY (chitin*) OR TITLE-ABS-KEY (phytate*) OR TITLE-ABS-KEY ("resistant starch") OR TITLE-ABS-KEY (dextrin*) OR TITLE-ABS-KEY (rs1) OR TITLE-ABS-KEY (rs2) OR TITLE-ABS-KEY (rs3) OR TITLE-ABS-KEY ("resistant oligosaccharide*") OR TITLE-ABS-KEY ("α-Galactoside*") OR TITLE-ABS-KEY (β-fructo-oligosaccharide*) OR TITLE-ABS-KEY (fos*) OR TITLE-ABS-KEY (α-galacto-oligosaccharide*) OR TITLE-ABS-KEY (gos*) OR TITLE-ABS-KEY (β-galacto-oligosaccharide*) OR TITLE-ABS-KEY (tos*) OR TITLE-ABS-KEY (xylo-oligosaccharide*) OR TITLE-ABS-KEY (xos*) OR TITLE-ABS-KEY (arabino-xylo-oligosaccharide*) OR TITLE-ABS-KEY (axos*) OR TITLE-ABS-KEY (polydextrose) OR TITLE-ABS-KEY ("Non-starch polysaccharide*") OR TITLE-ABS-KEY (cellulose) OR TITLE-ABS-KEY (hemicellulose*) OR TITLE-ABS-KEY (glucuronoxylan*) OR TITLE-ABS-KEY (heteroxylan*) OR TITLE-ABS-KEY (x?loglucan*) OR TITLE-ABS-KEY (mannan*) OR TITLE-ABS-KEY (galactomannan*) OR TITLE-ABS-KEY (glucomannan*) OR TITLE-ABS-KEY (galactoglucomannan*) OR TITLE-ABS-KEY (pectin*) OR TITLE-ABS-KEY (polygalacturonic) OR TITLE-ABS-KEY (gum*) OR TITLE-ABS-KEY (mucilage*) OR TITLE-ABS-KEY (arabinogalactan) OR TITLE-ABS-KEY (xanthan) OR TITLE-ABS-KEY (alginate*) OR TITLE-ABS-KEY (agar) OR TITLE-ABS-KEY (agar-agar) OR TITLE-ABS-KEY (carrageenan) OR TITLE-ABS-KEY (inulin) OR TITLE-ABS-KEY (fructan*) OR TITLE-ABS-KEY (β-glucan*)))) | ("Dietary Fiber"[MeSH Terms] OR "dietary fiber*"[Title/Abstract] OR "dietary fibre*"[Title/Abstract] OR "alimentary fiber*"[Title/Abstract] OR "alimentary fibre*"[Title/Abstract] OR (("Eating"[MeSH Terms] OR "eat"[Title/Abstract] OR "Diet"[MeSH Terms] OR "diet*"[Title/Abstract] OR "diet, food, and nutrition"[MeSH Terms] OR "nutri*"[Title/Abstract] OR "Food"[MeSH Terms] OR "food*"[Title/Abstract] OR ("intake"[Title/Abstract] OR "intake s"[Title/Abstract] OR "intakes"[Title/Abstract]) OR "Dietary Supplements"[MeSH Terms] OR "supplement*"[Title/Abstract] OR "consum*"[Title/Abstract]) AND ("fibre*"[Title/Abstract] OR "fiber*"[Title/Abstract] OR ("Dietary Fiber"[MeSH Terms] OR ("dietary"[Title/Abstract] AND "fiber"[Title/Abstract]) OR "Dietary Fiber"[Title/Abstract] OR "roughage"[Title/Abstract] OR "roughages"[Title/Abstract]) OR "Lignin"[MeSH Terms] OR ("Lignin"[MeSH Terms] OR "Lignin"[Title/Abstract] OR "lignins"[Title/Abstract] OR "lignin s"[Title/Abstract]) OR "Chitin"[MeSH Terms] OR "chitin*"[Title/Abstract] OR "phytate*"[Title/Abstract] OR "Resistant Starch"[MeSH Terms] OR "Resistant Starch"[Title/Abstract] OR "Dextrins"[MeSH Terms] OR "dextrin*"[Title/Abstract] OR "RS1"[Title/Abstract] OR "RS2"[Title/Abstract] OR "RS3"[Title/Abstract] OR "resistant oligosaccharide*"[Title/Abstract] OR "alpha galactoside*"[Title/Abstract] OR "beta fructo oligosaccharide*"[Title/Abstract] OR "fos"[Title/Abstract] OR "alpha galacto oligosaccharide*"[Title/Abstract] OR "gos"[Title/Abstract] OR "beta galacto oligosaccharide*"[Title/Abstract] OR "tos"[Title/Abstract] OR "Xylose"[MeSH Terms] OR "Xylose"[Title/Abstract] OR "xylo oligosaccharide*"[Title/Abstract] OR "xos"[Title/Abstract] OR "arabino xylo oligosaccharide*"[Title/Abstract] OR "axos*"[Title/Abstract] OR "polydextrose"[Supplementary Concept] OR ("polydextrose"[Supplementary Concept] OR "polydextrose"[Title/Abstract]) OR "non starch polysaccharide*"[Title/Abstract] OR "Cellulose"[MeSH Terms] OR ("Cellulose"[MeSH Terms] OR "Cellulose"[Title/Abstract] OR "celluloses"[Title/Abstract] OR "cellulosic"[Title/Abstract] OR "cellulosics"[Title/Abstract]) OR "hemicellulose*"[Title/Abstract] OR "glucuronoxylan"[Supplementary Concept] OR "glucuronoxylan*"[Title/Abstract] OR "heteroxylan*"[Title/Abstract] OR ("x"[Title/Abstract] AND "loglucan*"[Title/Abstract]) OR "Mannans"[MeSH Terms] OR "mannan*"[Title/Abstract] OR "galactomannan*"[Title/Abstract] OR "glucomannan*"[Title/Abstract] OR "galactoglucomannan*"[Title/Abstract] OR "Pectins"[MeSH Terms] OR "pectin*"[Title/Abstract] OR ("polygalacturonate"[Title/Abstract] OR "polygalacturonates"[Title/Abstract] OR "polygalacturonic"[Title/Abstract]) OR "Plant Gums"[MeSH Terms] OR "gum"[Title/Abstract] OR "Plant Mucilage"[MeSH Terms] OR "mucilage*"[Title/Abstract] OR "arabinogalactan"[Supplementary Concept] OR ("arabinogalactan"[Supplementary Concept] OR "arabinogalactan"[Title/Abstract] OR "arabinogalactans"[Title/Abstract]) OR "xanthan gum"[Supplementary Concept] OR ("xanthan gum"[Supplementary Concept] OR "xanthan gum"[Title/Abstract] OR "xanthan"[Title/Abstract] OR "xanthans"[Title/Abstract]) OR "Alginates"[MeSH Terms] OR "alginate*"[Title/Abstract] OR "Agar"[MeSH Terms] OR ("Agar"[MeSH Terms] OR "Agar"[Title/Abstract]) OR "agar-agar"[Title/Abstract] OR "Carrageenan"[MeSH Terms] OR ("Carrageenan"[MeSH Terms] OR "Carrageenan"[Title/Abstract] OR "carrageenans"[Title/Abstract]) OR "Inulin"[MeSH Terms] OR ("Inulin"[MeSH Terms] OR "Inulin"[Title/Abstract] OR "inuline"[Title/Abstract] OR "inulins"[Title/Abstract]) OR "Fructans"[MeSH Terms] OR "fructan*"[Title/Abstract] OR "beta-Glucans"[MeSH Terms] OR "beta glucan*"[Title/Abstract]))) |
| AND | | | |
| CLGI keywords | ((((((((((((((((((((((((((((((((((ALL=("low grade chronic inflammation")) OR ALL=("chronic low grade inflammation")) OR ALL=("systemic chronic inflammation")) OR ALL=("systemic low grade inflammation")) OR ALL=("subclinical inflammation")) OR ALL=("inflammat* *marker*")) OR ALL=("inflammat* mediator*")) OR ALL=("inflammat* index")) OR ALL=("inflammat* indices")) OR ALL=(citokine*)) OR ALL=(Interleukin*)) OR ALL=(Il-6)) OR ALL=(IL-1β)) OR ALL=(IL-10)) OR ALL=("Tumor necrosis factor-alpha")) OR ALL=(TNF-α)) OR ALL=(Interferon*)) OR ALL=(IFN*)) OR ALL=("Acute-phase protein*")) OR ALL=("C-reactive protein")) OR ALL=(CRP)) OR ALL=(C3)) OR ALL=("complement component 4")) OR ALL=("complement factor 3")) OR ALL=("third component of complement")) OR ALL=(C4)) OR ALL=("complement component 4")) OR ALL=("complement factor 4")) OR ALL=("fourth component of complement”)) OR ALL=("white blood cell count")) OR ALL=(WBC)) OR ALL=("white blood cell")) OR ALL=(neutrophil*)) OR ALL=(lymphocyte*)) OR ALL=(adiponectin) | ALL ("low grade chronic inflammation") OR ALL ("chronic low grade inflammation") OR ALL ("systemic chronic inflammation") OR ALL ("systemic low grade inflammation") OR ALL ("subclinical inflammation") OR ALL ("inflammat* *marker*") OR ALL ("inflammat* mediator*") OR ALL ("inflammat* index") OR ALL ("inflammat* indices") OR ALL (citokine*) OR ALL (interleukin*) OR ALL (il-6) OR ALL (il-1β) OR ALL (il-10) OR ALL ("Tumor necrosis factor-alpha") OR ALL (tnf-α) OR ALL (interferon*) OR ALL (ifn*) OR ALL ("Acute-phase protein*") OR ALL ("C-reactive protein") OR ALL (crp) OR ALL (c3) OR ALL ("complement component 4") OR ALL ("complement factor 3") OR ALL ("third component of complement") OR ALL (c4) OR ALL ("complement component 4") OR ALL ("complement factor 4") OR ALL ("fourth component of complement") OR ALL ("white blood cell count") OR ALL (wbc) OR ALL ("white blood cell") OR ALL (neutrophil*) OR ALL (lymphocyte*) OR ALL (adiponectin)) | ("low grade chronic inflammation"[All Fields] OR "chronic low grade inflammation"[All Fields] OR "systemic chronic inflammation"[All Fields] OR "systemic low grade inflammation"[All Fields] OR "subclinical inflammation"[All Fields] OR "Inflammation Mediators"[MeSH Terms] OR "inflammation marker*"[All Fields] OR "inflammation mediator*"[All Fields] OR "inflammation index"[All Fields] OR "inflammation indices"[All Fields] OR "Cytokines"[MeSH Terms] OR "citokine*"[All Fields] OR "Interleukins"[MeSH Terms] OR "interleukin*"[All Fields] OR "Interleukin-6"[MeSH Terms] OR ("Interleukin-6"[MeSH Terms] OR "Interleukin-6"[All Fields] OR "il 6"[All Fields]) OR "Interleukin-1beta"[MeSH Terms] OR ("Interleukin-1beta"[MeSH Terms] OR "Interleukin-1beta"[All Fields] OR ("il"[All Fields] AND "1beta"[All Fields]) OR "il 1beta"[All Fields]) OR "Interleukin-10"[MeSH Terms] OR ("Interleukin-10"[MeSH Terms] OR "Interleukin-10"[All Fields] OR "il 10"[All Fields]) OR "Tumor Necrosis Factor-alpha"[MeSH Terms] OR ("Tumor Necrosis Factor-alpha"[MeSH Terms] OR ("tumor"[All Fields] AND "necrosis"[All Fields] AND "factor alpha"[All Fields]) OR "Tumor Necrosis Factor-alpha"[All Fields] OR ("tnf"[All Fields] AND "alpha"[All Fields]) OR "tnf alpha"[All Fields]) OR "Interferons"[MeSH Terms] OR "interferon*"[All Fields] OR "ifn"[All Fields] OR "Acute-Phase Proteins"[MeSH Terms] OR "acute phase protein*"[All Fields] OR "c reactive protein"[MeSH Terms] OR "c reactive protein"[All Fields] OR ("curr res psychol"[Journal] OR "crp"[All Fields]) OR "Complement C3"[MeSH Terms] OR "C3"[All Fields] OR "complement factor 3"[All Fields] OR "Complement C4"[MeSH Terms] OR "complement component 4"[All Fields] OR "C4"[All Fields] OR "Leukocyte Count"[MeSH Terms] OR "white blood cell count"[All Fields] OR ("Leukocyte Count"[MeSH Terms] OR ("leukocyte"[All Fields] AND "count"[All Fields]) OR "Leukocyte Count"[All Fields] OR "wbc"[All Fields]) OR "Leukocytes"[MeSH Terms] OR "white blood cell"[All Fields] OR "Neutrophils"[MeSH Terms] OR "neutrophil*"[All Fields] OR "Lymphocytes"[MeSH Terms] OR "lymphocyte*"[All Fields] OR "Adiponectin"[MeSH Terms] OR ("Adiponectin"[MeSH Terms] OR "Adiponectin*"[All Fields])) |
| AND | | | |
| Population keywords | ((TS=(child*)) OR TS=(infant)) OR TS=(adolescent*) | (TITLE-ABS-KEY (child*) OR TITLE-ABS-KEY (infant) OR TITLE-ABS-KEY (adolescent*)))) | ("Child"[MeSH Terms] OR "child*"[Title/Abstract] OR "Infant"[MeSH Terms] OR "Infant"[Title/Abstract] OR "Adolescent"[MeSH Terms] OR "adolescent*"[Title/Abstract]) |
| AND | | | |
| Study design keywords ^[1]^ | TS=(randomised OR randomized OR randomisation OR randomization OR placebo* OR (random* AND (allocat* OR assign*)) OR (blind* AND (single OR double OR treble OR triple))) | TITLE-ABS-KEY ({Clinical-trial} OR {controlled-trial} OR randomi* OR randomly OR (random W/4 (allocat* OR distribut* OR assign*)) OR {placebo} OR {trial} OR {groups} OR {subgroups}) OR TITLE (rct)) | (("randomized controlled trial"[Publication Type] OR "controlled clinical trial"[Publication Type] OR "randomized"[Title/Abstract] OR "placebo"[Title/Abstract] OR "drug therapy"[MeSH Subheading] OR "randomly"[Title/Abstract] OR ("trial"[Title/Abstract] OR "groups"[Title/Abstract])) NOT ("animals"[MeSH Terms] NOT "humans"[MeSH Terms])) |
| **HITS** | **893** | **725** | **161** |

**Supplementary Table 1**: Search strategy in Web of Science, PubMed and Scopus databases.

**Supplementary Table 2**

| **ID** **Search** **Hits**  #1 MeSH descriptor: [Dietary Fiber] explode all trees 2710  #2 (dietary NEXT fiber*):ti,ab,kw OR (dietary NEXT fibre*):ti,ab,kw OR (alimentary NEXT fiber*):ti,ab,kw OR (alimentary NEXT fibre*):ti,ab,kw 4067  #3 #1 OR #2 4511  #4 MeSH descriptor: [Eating] explode all trees 5066  #5 MeSH descriptor: [Diet] explode all trees 26489  #6 MeSH descriptor: [Diet, Food, and Nutrition] explode all trees 76846  #7 MeSH descriptor: [Food] explode all trees 48969  #8 MeSH descriptor: [Dietary Supplements] explode all trees 19802  #9 (eat*):ti,ab,kw OR (diet*):ti,ab,kw OR (nutri*):ti,ab,kw OR (food*):ti,ab,kw OR (intake):ti,ab,kw OR (supplement*):ti,ab,kw OR (consum*):ti,ab,kw 298071  #10 #4 OR #5 OR #6 OR #7 OR #8 OR #9 308984  #11 MeSH descriptor: [Lignin] explode all trees 12  #12 MeSH descriptor: [Chitin] explode all trees 294  #13 MeSH descriptor: [Resistant Starch] explode all trees 39  #14 MeSH descriptor: [Dextrins] explode all trees 411  #15 MeSH descriptor: [Xylose] explode all trees 57  #16 MeSH descriptor: [Cellulose] explode all trees 1102  #17 MeSH descriptor: [Mannans] explode all trees 336  #18 MeSH descriptor: [Pectins] explode all trees 168  #19 MeSH descriptor: [Plant Gums] explode all trees 1079  #20 MeSH descriptor: [Plant Mucilage] explode all trees 4  #21 MeSH descriptor: [Alginates] explode all trees 371  #22 MeSH descriptor: [Agar] explode all trees 33  #23 MeSH descriptor: [Carrageenan] explode all trees 54  #24 MeSH descriptor: [Inulin] explode all trees 334  #25 MeSH descriptor: [Fructans] explode all trees 364  #26 MeSH descriptor: [beta-Glucans] explode all trees 321  #27 (fibre*):ti,ab,kw OR (fiber*):ti,ab,kw OR (roughage):ti,ab,kw OR (lignin):ti,ab,kw OR (chitin*):ti,ab,kw OR (phytate*):ti,ab,kw OR ("resistant starch"):ti,ab,kw OR (dextrin*):ti,ab,kw OR (RS1):ti,ab,kw OR (RS2):ti,ab,kw OR (RS3):ti,ab,kw OR (resistant NEXT oligosaccharide*):ti,ab,kw OR (α-Galactoside*):ti,ab,kw OR (β-fructo-oligosaccharide*):ti,ab,kw OR (FOS*):ti,ab,kw OR (α-galacto-oligosaccharide*):ti,ab,kw OR (GOS*):ti,ab,kw OR (β-galacto-oligosaccharide*):ti,ab,kw OR (TOS*):ti,ab,kw OR (xylose):ti,ab,kw OR (xylo-oligosaccharide*):ti,ab,kw OR (XOS*):ti,ab,kw OR (arabino-xylo-oligosaccharide*):ti,ab,kw OR (AXOS*):ti,ab,kw OR (polydextrose):ti,ab,kw OR (Non-starch NEXT polysaccharide*):ti,ab,kw OR (cellulose):ti,ab,kw OR (hemicellulose*):ti,ab,kw OR (glucuronoxylan*):ti,ab,kw OR (heteroxylan*):ti,ab,kw OR (x?loglucan*):ti,ab,kw OR (mannan*):ti,ab,kw OR (galactomannan*):ti,ab,kw OR (glucomannan*):ti,ab,kw OR (galactoglucomannan*):ti,ab,kw OR (pectin*):ti,ab,kw OR (polygalacturonic):ti,ab,kw OR (gum*):ti,ab,kw OR (mucilage*):ti,ab,kw OR (arabinogalactan):ti,ab,kw OR (xanthan):ti,ab,kw OR (alginate*):ti,ab,kw OR (agar):ti,ab,kw OR (agar-agar):ti,ab,kw OR (carrageenan):ti,ab,kw OR (inulin):ti,ab,kw OR (fructan*):ti,ab,kw OR (β-glucan*):ti,ab,kw 43852  #28 #11 OR #12 OR #13 OR #14 OR #15 OR #16 OR #17 OR #18 OR #19 OR #20 OR #21 OR #22 OR #23 OR #24 OR #25 OR #26 OR #27 44947  #29 #10 AND #28 15622  #30 #3 OR #29 15780  #31 MeSH descriptor: [Inflammation Mediators] explode all trees 16135  #32 MeSH descriptor: [Cytokines] explode all trees 27451  #33 MeSH descriptor: [Interleukins] explode all trees 8917  #34 MeSH descriptor: [Interleukin-6] explode all trees 4360  #35 MeSH descriptor: [Interleukin-1beta] explode all trees 642  #36 MeSH descriptor: [Interleukin-10] explode all trees 1139  #37 MeSH descriptor: [Tumor Necrosis Factor-alpha] explode all trees 4366  #38 MeSH descriptor: [Interferons] explode all trees 7265  #39 MeSH descriptor: [Acute-Phase Proteins] explode all trees 9084  #40 MeSH descriptor: [C-Reactive Protein] explode all trees 6340  #41 MeSH descriptor: [Complement C3] explode all trees 335  #42 MeSH descriptor: [Complement C4] explode all trees 111  #43 MeSH descriptor: [Leukocyte Count] explode all trees 6457  #44 MeSH descriptor: [Leukocytes] explode all trees 12649  #45 MeSH descriptor: [Neutrophils] explode all trees 1788  #46 MeSH descriptor: [Lymphocytes] explode all trees 7129  #47 MeSH descriptor: [Adiponectin] explode all trees 1154  #48 ("low grade chronic inflammation") OR ("chronic low grade inflammation") OR ("systemic chronic inflammation") OR ("systemic low grade inflammation") OR ("subclinical inflammation") OR (inflammaiton NEXT marker*) OR (inflammation NEXT mediator*) OR (inflammation index) OR (inflammation indices) OR (citokine*) OR (interleukin*) OR (il-6) OR (il-1β) OR (il-10) OR (TNF-α) OR (interferon*) OR (IFN*) OR (acute-phase NEXT protein*) OR ("C-reactive protein") OR (CRP) OR (C3) OR ("complement factor 3") OR (C4) OR ("component factor 4") OR ("white blood cell count") OR (WBC) OR ("white blood cell") OR (neutrophil*) OR (lymphocyte*) OR (adiponectin) 121418  #49 #31 OR #32 OR #33 OR #34 OR #35 OR #36 OR #37 OR #38 OR #39 OR #40 OR #41 OR #42 OR #43 OR #44 OR #45 OR #46 OR #47 OR #48 144238  #50 MeSH descriptor: [Child] explode all trees 81197  #51 MeSH descriptor: [Adolescent] explode all trees 136261  #52 MeSH descriptor: [Infant] explode all trees 45750  #53 (child*):ti,ab,kw OR (infant):ti,ab,kw OR (adolescent*):ti,ab,kw 335429  #54 #50 OR #51 OR #52 OR #53 335429  #55 #30 AND #49 1697  #56 #55 AND #54 269 // **251 trials** |
| --- |

**Supplementary Table 2:** Search strategy in CENTRAL database

**Supplementary Table 3**

| Author, year | Title of the protocol | Number register protocol | Register where found | Expected completation date or current phase | COUNTRY | PARTICIPANTS | | INTERVENTION | | GROUPS | | OUTCOMES |
| --- | --- | --- | --- | --- | --- | --- | --- | --- | --- | --- | --- | --- |
|  |  |  |  |  |  | age | Health status | Duration | Design | I  (source and dose of fiber (g)) | C |  |
| Lamuela-Reventós RM et al. , 2023 | Peanut Consumption on Cognitive, Weight, and Inflammation | NCT06127511 | ClinicalTrials.gov | 12/2024 | Spain | Adolescents | Healty | 6 months | two-arm parallel cluster-randomized controlled trial | 25 g of whole skin roasted peanuts. The protocol states that peanuts are a source of fiber, yet no information is provided regarding the quantity of fibre present so far. | - | Adiponectin, il-6, il-10 |
| Khadilkar A et al., 2023 | Proof of Concept Study to evaluate the effect of Iron and Prebiotic Supplementation in Children | CTRI/2023/03/050188 | ICTRP | Recruitment complete, intervention 3/2024 | India | 3-7 year old children | Iron defincy | 3 months | Single Arm Study Method computer generated randomization | Suplemmentation with Prebiotic- 3.41 g (but other components, check for eligibility) | - | CRP |

**Supplementary Table 3:** Ongoing studies potentially relevant for inclusion in any future update of the SR

**Supplementary Figure 1**

**Supplementary Figure 1:** PRISMA flow chart study selection

**Supplementary Table 4**

| **Author**  **Publication Year** |  |
| --- | --- |
|  | **Baseline values of serum inflammatory markers for CLGI** |
| Feruś et al. 2018  ^[52]^ | \|  \| Synergy 1 Group \| Placebo Group \| \| --- \| --- \| --- \| \| CRP (mg/L) \| 1.4±0.8 \| 1 ±0.8 \| \| White Blood Cell (10^3^/mm^3^) \| 6.29±1.64 \| 6.59±1.64 \| |
| Drabińska et al.  2019  ^[53]^ | \|  \| Synergy 1 Group \| Placebo Group \| \| --- \| --- \| --- \| \| IL-1β (pg/mL) \| 1.74(1.63-1.82) \| 1.74(1.59-1.88) \| \| IL-1ra (pg/mL) \| 156.3(136.4-187.5) \| 247.3(139.5-690.9) \| \| IL-6 (pg/mL) \| 0.89(0.73-1.13) \| 0.5(0.41-1.1) \| \| IL-8 (pg/mL) \| 3.74(3.34-4.12) \| 4.22(3.57-4.79) \| \| IL-10 (pg/mL) \| 3.1(2.71-3.3) \| 3.07(2.95-3.26) \| \| TNF-α (pg/mL) \| 0.95(0.53-1.24) \| 0.91(0.56-1.17) \| |
| Nicolucci et al.  2017  ^[76]^ | \|  \| Prebiotics Group \| Placebo Group \| \| --- \| --- \| --- \| \| CRP (mg/L) \| 2.3±0.4 \| 2.4±0.5 \| \| IFNγ (pg/mL) \| 16.58±3.98 \| 11.89±3.02 \| \| IL-4 (pg/mL) \| 2.27±0.78 \| 3.46±1.97 \| \| IL-1β (pg/mL) \| 0.98±0.11 \| 1.07±0.21 \| \| IL-6 (pg/mL) \| 0.76±0.16 \| 0.51±0.12 \| \| IL-10 (pg/mL) \| 3.72±0.65 \| 4.35±1.19 \| \| TNF-α (pg/mL) \| 4.26±0.46 \| 3.93±0.45 \| |
| Ho et al.  2019  ^[77]^ | \|  \| Prebiotics Group \| Placebo Group \| \| --- \| --- \| --- \| \| IFNγ (pg/mL) \| 444.13±1792.58 \| 20.57±44.7 \| \| IL-6 (pg/mL) \| 43.97±148.59* \| 7.35±8.95* \| \| IL-10 (pg/mL) \| 15.1±36.17 \| 12.98±17.91 \| \| TNF-α (pg/mL) \| 16.08±52.33* \| 3.89±1.75 \| |
| Visuthranukul et al.  2022  ^[57]^ | \|  \| Inulin Group \| Placebo Group \| \| --- \| --- \| --- \| \| IL-1β (pg/mL) \| 0.31±108.2 \| 0.33±110.6 \| \| IL-6 (pg/mL) \| 1.1±80 \| 0.99±108.2 \| \| TNF-α (pg/mL) \| 15.7±49.2 \| 15.2±48.8 \| |
| Paganini, Uyoga, Cercamondi et al.  2017  ^[65]^ | \|  \| Intervention Group \| Control Group \| \| --- \| --- \| --- \| \| CRP (mg/L) \| 0.7 (0.3 -1.7) \| 0.6(0.3-2.2) \| |
| Mikulic et al.  2024  ^[66]^ | \|  \| Intervention Group \| Control Group \| \| --- \| --- \| --- \| \| CRP (mg/L) \| 1.16(0.05-4.21) \| 1.16(0.25-3.41) \| |
| Paganini, Uyoga, Kortman et al.  2017  ^[67]^ | \|  \| Intervention Group \| Control Group \| \| --- \| --- \| --- \| \| CRP (mg/L) \| 1.2(0.5-6.5) \| 1.2(0.5-3.2) \| |
| Raes et al.  2010  ^[54^ | NR |
| Van den Berg et al.  2013  ^[55]^ | NR |
| Zheng et al.  2006  ^[58]^ | \|  \| Intervention Group \| Control Group \| \| --- \| --- \| --- \| \| TNF-α (ng/mL) \| 1.44±0.77 \| 1.40±0.97 \| \| IL-2 (ng/mL) \| 183.9±105 \| 172.8±67.6 \| \| IL-6 (ng/mL) \| 91.0±54.4 \| 84.9±56.7 \| |
| López-Velázquez et al.  2015  ^[73]^ | \|  \| Intervention Group \| Control Group \| \| --- \| --- \| --- \| \| CRP (mg/L) \| 3.29±0.7* \| 2.97±0.4* \| |
| Henao et al.  2018  ^[64]^ | NR |
| González et al.  2021  ^[74]^ | \|  \| Intervention Group \| Control Group \| \| --- \| --- \| --- \| \| IL-6 (pg/mL) \| 3.32 (1.24-5.96) \| 2.1 (1.07-4.1) \| |
| Fatahi et al.  2022  ^[59]^ | \|  \| Intervention Group \| Placebo Group \| \| --- \| --- \| --- \| \| Adiponectin (ng/mL) \| 7.55±4.2 \| 10.09±5.32 \| |
| Bseikri et al.  2018  ^[72]^ | \|  \| Intervention Group \| Placebo Group \| \| --- \| --- \| --- \| \| hs-CRP (mg/L) \| 2.47±1.91 \| 3.71±5.79 \| \| Adiponectin (ng/mL) \| 1807±564.3 \| 2366±1980 \| |
| Mietus-Snyder et al.  2020  ^[68]^ | \|  \| Intervention Group \| Control Group \| \| --- \| --- \| --- \| \| CRP (nmol/L) \| 28.6±22.8 \| 57.1±30.5 \| \| Adiponectin (ng/mL) \| 1296±587) \| 1313±588 \| |
| Anaya-Loyola  2020  ^[75]^ | \|  \| Intervention Group \| Control Group \| \| --- \| --- \| --- \| \| CRP (mg/L) \| 2.892±1.304 \| 2.413±0.965 \| \| IL-6 (pg/mL) \| 14.704±3.829 \| 13.675±5.182 \| |
| Da Silva et al.  2020  ^[63]^ | \|  \| Intervention Group \| Control Group \| \| --- \| --- \| --- \| \| TNF-α (pg/mL) \| 7.6±1.8* \| 6.8±2.7* \| \| IL-6 (pg/mL) \| 2.7±0.8 \| 2.8±1.2 \| |
| Zambrana et al.  2021  ^[61]^ | NR |
| Madsen et al.  2024  ^[56]^ | \|  \| Group starting with whole grains \| Group strarting with refined grains \| \| --- \| --- \| --- \| \| CRP (mg/L) \| 0.31 (0.23-0.72) \| 0.44(0.2-0.82) \| \| IL-6 (pmol/L) \| 0.88(0.59-1.16) \| 0.9(0.56-1.52) \| |
| Vaz-Tostes et al.  2014  ^[62]^ | \|  \| Intervention Group \| Control Group \| \| --- \| --- \| --- \| \| IL-4 (pg/mL) \| 1.17±0.37 \| 0.736±0.182 \| \| IL-6 (pg/mL) \| 1.086±0.24 \| 1.751±0.399 \| \| IL-10 (pg/mL) \| 3.726±0.527 \| 5.292±0.72 \| \| TNF-α (pg/mL) \| 12.51±0.953* \| 24.164±2.26* \| |
| Eisner et al.  2020  ^[69]^ | \|  \| Intervention Group \| Control Group \| \| --- \| --- \| --- \| \| CRP (mg/L) \| 0.003±0.004 \| 0.002±0.002 \| \| Adiponectin (µg/mL) \| 4.8±2.3 \| 4.6±2.1 \| |
| Langkamp-Henken et al.  2012  ^[70]^ | \|  \| Intervention Group \| Control Group \| \| --- \| --- \| --- \| \| CRP (mg/L) \| 1.1±0.3 \| 0.6±0.1 \| |
| Hajihashemi et al.  2014  ^[60]^ | \|  \| Intervention Group \| Control Group \| \| --- \| --- \| --- \| \| CRP (mg/L) \| 2.52±1.96 \| 1.65±1.43 \| |
| Hasson et al.  2012  ^[71]^ | \|  \| African Americans \| Latinos \| \| --- \| --- \| --- \| \| Adiponectin (µg/mL) \| 17.2±1.3 \| 18.9±11.1 \| \| IL-8 (pg/mL) \| 2.6±0.3* \| 4.1±0.5* \| \| TNF-α (pg/mL) \| 6.4±0.5* \| 13.5±1.1* \| |

**Supplementary Table 4:** Baseline levels of serum inflammatory markers observed in pediatric populations of the included studies. Values exceeding the threshold or cutoff point indicative of inflammation are marked with an asterisk (*).

**Supplementary Table 5**

| **Covariate** | **Coefficient (β)** | **95% Confidence Interval** | **Standard Error** | **p-value** |
| --- | --- | --- | --- | --- |
| **Intercept** | -2.677 | (-6.221, 0.868) | 1.808 | 0.139 |
| **Grams of additional fiber in the intervention group/day** | 0.169 | (-0.013, 0.352) | 0.093 | 0.069 |
| **Duration of the intervention (weeks)** | 0.038 | (-0.132, 0.208) | 0.087 | 0.659 |
| **Type of fiber** |  |  |  |  |
| - Fiber-rich food or product | Reference group |  |  |  |
| - Fiber supplementation | -1.358 | (-2.396, -0.320) | 0.530 | 0.010* |
| **Health condition** |  |  |  |  |
| - Healthy | Reference group |  |  |  |
| - Chronic disease | -0.831 | (-2.791, 1.129) | 1.000 | 0.406 |
| - Overweight or obesity | -1.016 | (-3.186, 1.154) | 0.525 | 0.725 |
| **Baseline CRP (mg/L)** |  |  |  |  |
| - <3 | Reference group |  |  |  |
| - >3 | 0.112 | (-0.917, 1.142) | 0.525 | 0.830 |
| **Omnibus p-value** |  |  |  | 0.009* |

**Supplementary Table 5:** Meta-regression model evaluating the association between dietary fiber interventions and changes in CRP levels. The table presents the estimated coefficients (β), 95% confidence intervals (CI), standard errors, and p-values for each covariate included in the model. The covariates analyzed were grams of additional fiber in the intervention group per day (continuous), duration of the intervention in weeks (continuous), type of fiber intervention, health condition of participants, and baseline CRP levels. Statistically significant p-values (<0.05) are marked with an asterisk (*).

**Supplementary Figure 2**

**Supplementary Figure 2:** Sensitivity analysis for CRP. **A:** Meta-analysis after excluding Nicolucci et al.^[68]^ resulted in a no longer significant reduction of CRP. **B:** Meta-analysis after excluding Anaya-Loyola et al.^[67]^, resulted in greater magnitude of the effect.

**Supplementary Figure 3**


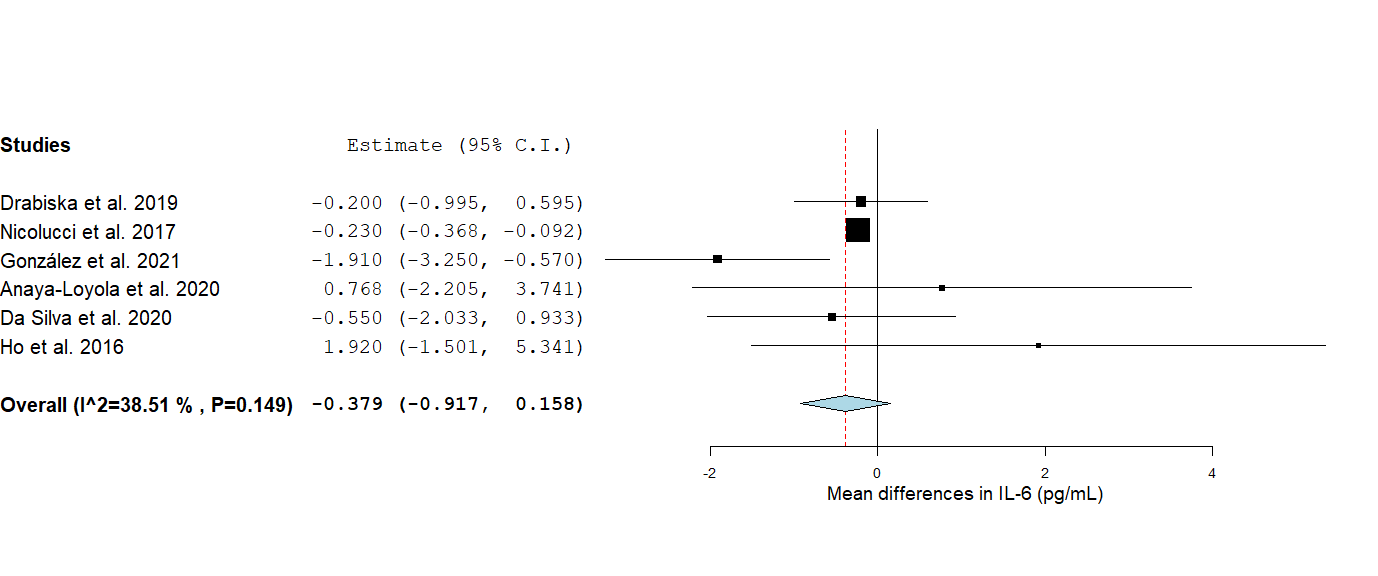


**Supplementary Figure 3:** Sensitivity analysis for IL-6. Meta-analysis after excluding outliers (Vaz-Tostes et al. ^[54]^) showed that interventions with fiber tended to decrease IL-6 concentrations, although not significantly (MD: -0.379; CI: -0.917, 0.158; I2 =38.5%).

**Supplementary Figure 4**


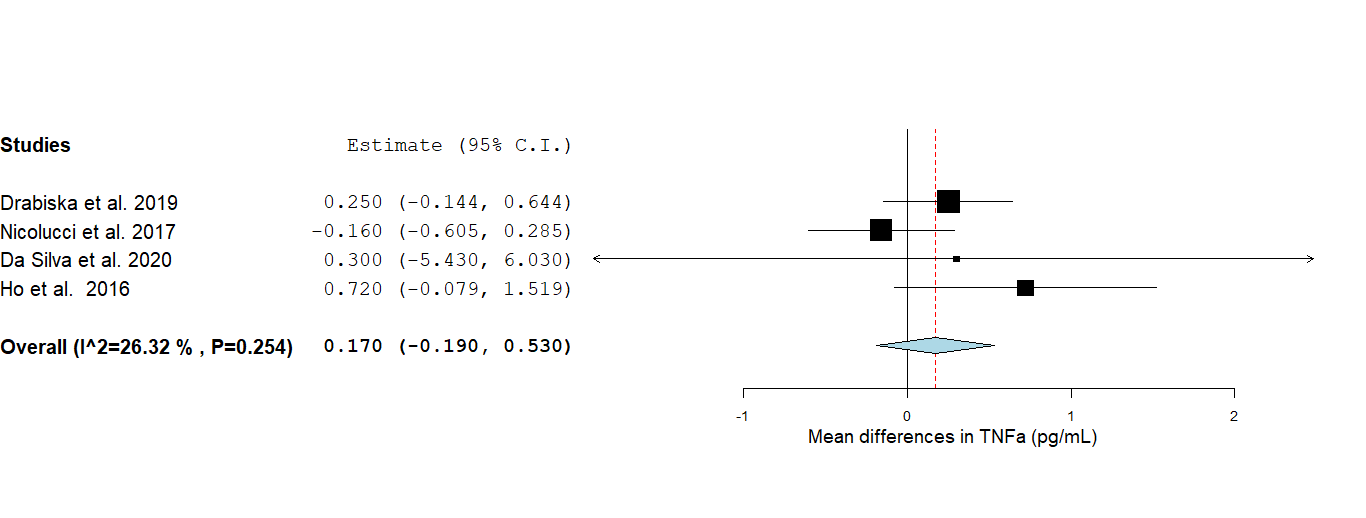


**Supplementary Figure 4:** Sensitivity analysis for TNFα. Meta-analysis after excluding outliers (Vaz-Tostes et al. ^[54]^) resulted in a lower increase of TNFα, still not significant (MD: 0.17; CI: -0.19, 0.53).

**Supplementary Table 6**

| (Study Author, Year) | Overall | Eligibility Criteria (1) | 2 | 3 | 4 | 5 | 6 | 7 | 8 | 9 | 10 | 11 |
| --- | --- | --- | --- | --- | --- | --- | --- | --- | --- | --- | --- | --- |
| (Anaya-Loyola et al., 2020) | 7/10 | X | X | X | X | X | X | 0 | 0 | 0 | X | X |
| (Bseikri et al., 2018) | 7/10 | X | X | X | X | 0 | 0 | 0 | X | X | X | X |
| (Feruś et al., 2018) | 9/10 | X | X | X | X | X | X | 0 | X | X | X | X |
| (Da Silva et al., 2020) | 7/10 | X | X | X | 0 | X | X | 0 | 0 | X | X | X |
| (Drabińska et al., 2019) | 9/10 | X | X | X | X | X | X | 0 | X | X | X | X |
| (Zambrana et al., 2021) | 6/10 | X | X | X | X | 0 | 0 | 0 | 0 | 0 | X | X |
| (Ho et al., 2019) | 9/10 | X | X | X | X | X | X | 0 | X | X | X | X |
| (López-Velázquez et al., 2015) | 8/10 | X | X | X | X | X | X | X | 0 | 0 | X | X |
| (Madsen et al., 2024) | 10/10 | X | X | X | X | 0 | 0 | X | X | X | X | X |
| (Raes et al., 2010) | 8/10 | X | X | X | 0 | X | X | 0 | X | X | X | X |
| (Visuthranukul et al., 2022) | 7/10 | X | X | X | X | X | X | 0 | X | X | 0 | 0 |
| (Paganini, Uyoga, Cercamondi, et al., 2017) | 9/10 | X | X | X | X | X | X | 0 | X | X | X | X |
| (Van den Berg et al., 2013) | 10/10 | X | X | X | X | X | X | X | X | X | X | X |
| (Zheng et al., 2006) | 10/10 | X | X | X | X | X | X | X | X | X | X | X |
| (Henao et al., 2018) | 8/10 | X | X | X | X | X | X | X | 0 | 0 | X | X |
| (González et al., 2021) | 9/10 | X | X | X | X | X | X | 0 | X | X | X | X |
| (Fatahi et al., 2022) | 10/10 | X | X | X | X | X | X | X | X | X | X | X |
| (Vaz-Tostes et al., 2014) | 4/10 | X | 0 | 0 | X | 0 | 0 | 0 | 0 | X | X | X |
| (Mikulic et al., 2024) | 8/10 | X | X | X | 0 | X | 0 | 0 | X | X | X | X |
| (Paganini et al., 2017) | 8/10 | X | X | 0 | X | X | X | 0 | X | X | X | X |
| (Nicolucci et al., 2017) | 10/10 | X | X | X | X | X | X | X | X | X | X | X |
| (Langkamp-Henken et al., 2012) | 8/10 | X | X | X | X | 0 | 0 | X | X | X | X | X |
| (Mietus-Snyder et al., 2020) | 6/10 | X | X | X | 0 | 0 | 0 | 0 | X | X | X | X |
| (Eisner et al., 2020) | 5/10 | X | X | X | 0 | 0 | 0 | 0 | 0 | 0 | X | X |
| (Hajihashemi et al., 2014) | 6/10 | X | X | X | 0 | 0 | 0 | 0 | X | X | X | X |
| (Hasson et al., 2012) | 6/10 | X | X | X | X | 0 | 0 | 0 | 0 | X | X | X |

**Supplementary Table 6:** PEDro scale quality assessment

**Supplementary Figure 5**


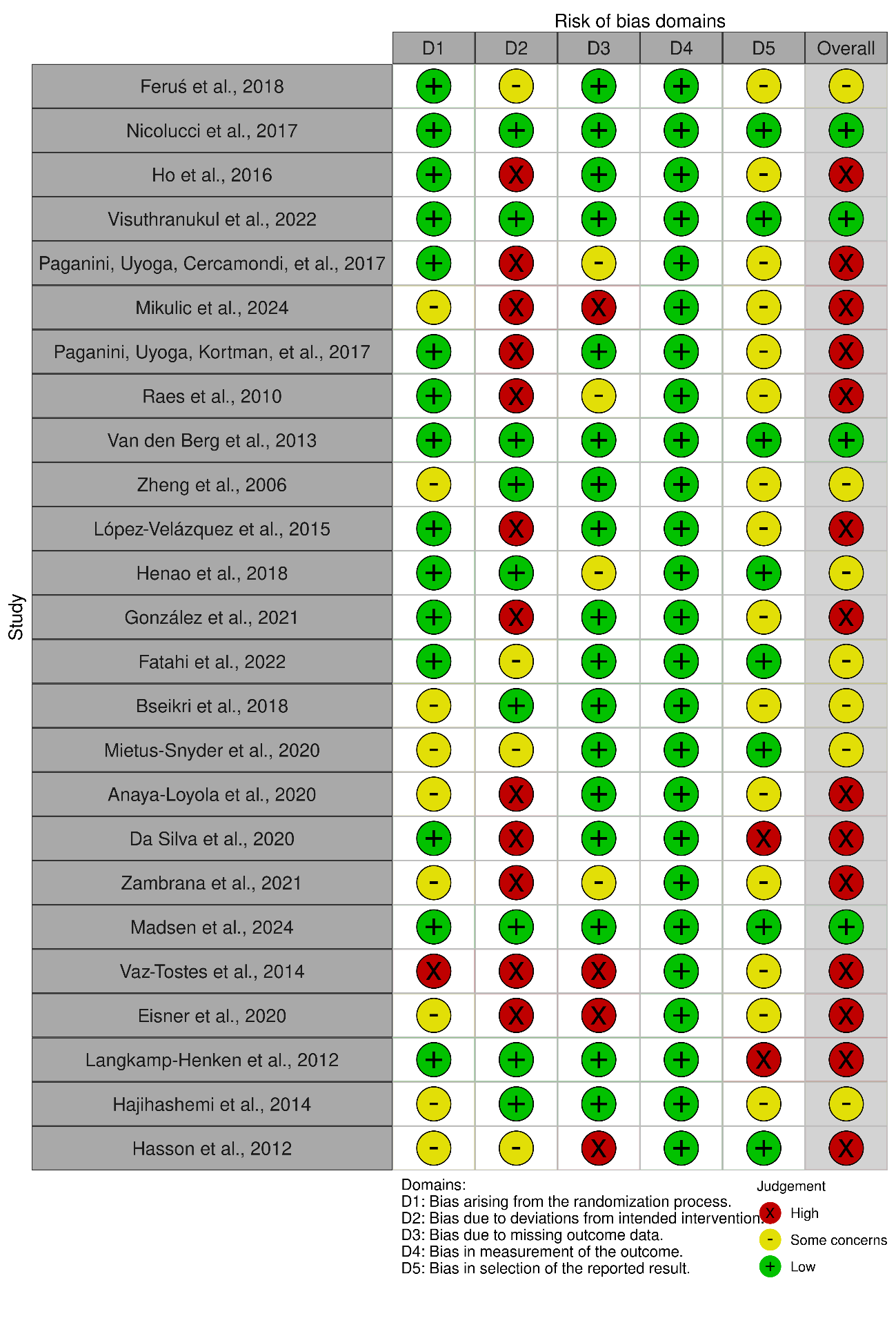

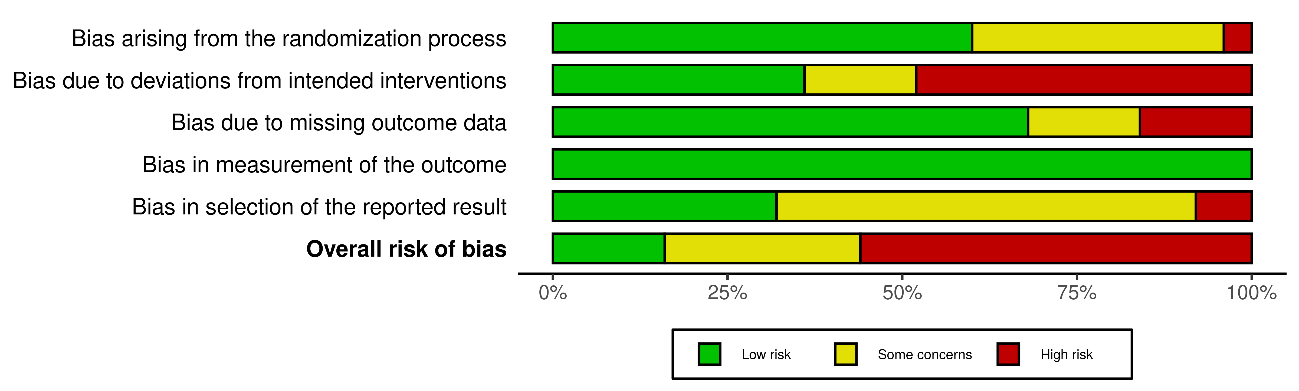


**Supplementary Figure 5:** Risk of bias assessment RoB2 tool
